# Effectiveness of multi-component, dynamic begging displays as honest signals of nestling hunger

**DOI:** 10.1098/rsos.260413

**Published:** 2026-10-14

**Authors:** Tomas Redondo, Daniel Parejo-Pulido

**Affiliations:** ^1^ Estación Biológica de Doñana (EBD), CSIC, Sevilla, Spain; ^2^ Instituto de Investigación en Recursos Cinegéticos (IREC), CSIC-UCLM-JCCM, Ciudad Real, Spain

**Keywords:** parent-offspring communication, nestling begging, honest signalling, pooling equilibria, multi-component signals

## Abstract

Parent–offspring communication depends on offspring signals that help parents assess need and allocate care. Nestling begging is often treated as a graded, information-rich and therefore honest signal of hunger, as predicted by costly signalling models. We quantified how effectively visual and acoustic begging components encode food deprivation in spotless starling (*Sturnus unicolor*) nestlings using field observations and a controlled laboratory experiment. We combined random-regression models with information-theoretic measures of uncertainty reduction and receiver-based machine-learning decoding. In wild broods, begging order and maximum postural intensity increased with deprivation time, yet posture and calling together reduced uncertainty about deprivation by less than 5%. Distinct postural levels overlapped widely among deprivation states and followed a stereotyped sequence—rapid escalation to maximum intensity followed by relaxation—yielding poor resolution of intermediate hunger. Adding components improved decoding only marginally beyond the first few cues. Parental allocation was correspondingly rapid, although display duration could provide additional information once the full bout was perceived. Discrete coding analyses confirmed that simple postural schemes were most consistent and that begging reliably distinguished only coarse, extreme hunger states. Overall, the results support partially informative (pooling) or attention-seeking signalling models in which parents rely on a small set of salient cues within a multi-component display.

## 1. Introduction

A key question in animal communication is whether signals convey honest information [1], enabling receivers to assess cryptic aspects of signallers that matter to them [2]. Traditional models implicitly assumed that signallers and receivers rely on similar coding schemes [3]. Although signals can carry information, their primary function is to influence receivers rather than inform them [4,5], and communication need not be honest [6]. For example, young birds (and other young animals) produce complex begging displays to solicit care from parents [7], and these displays are widely viewed as honest indicators of need, with parents using signal intensity to guide provisioning and allocation decisions [8]. Yet begging is supposedly also an influential signal shaped by evolutionary parent–offspring conflict over resource allocation, with offspring selected to demand more than parents are willing to provide [9,10].

Signalling theory in the context of parent–offspring communication and begging gained prominence following Godfray's seminal model [11,12], which predicts a stable, honest equilibrium in which parents obtain accurate information about offspring's need through a one-to-one mapping between need and begging intensity. In early interpretations of these models, stable honesty was often linked to the existence of substantial signalling costs at equilibrium. However, recent signalling theory has shifted the emphasis from realized equilibrium costs to incentive structure and underlying trade-offs [13–15]. Under this view, honesty does not require signals to be intrinsically costly once the honest equilibrium is reached; rather, it is maintained when alternative signalling strategies are sufficiently disadvantageous for individuals in different states or conditions. This perspective is consistent with empirical evidence from begging, where support for substantial intrinsic costs—particularly in birds—is scant [16,17], without implying that begging cannot function as an honest signal.

A separate question is how much information begging actually conveys. Godfray's signalling model assumes that nestling begging is highly informative [18,19] and focuses on a separating equilibrium, in which different states produce distinct signals and receivers obtain complete information [20]. However, such highly informative equilibria may be evolutionarily unlikely [13,21–24]. Young birds and other animals increase their solicitation when food-deprived—a major, though not exclusive, correlate of need [25,26]. Thus, there is a broad consensus that begging signals convey some information to parents about the offspring's state [21,27,28]. However, begging is highly plastic and strongly influenced by factors beyond nutritional condition, including volatile contextual variables, such as nestling experience [29], feeding history [30], social context [31,32], temperature [33] and perceived predation risk (e.g. [34,35]), raising doubts about the precision and reliability of the information conveyed. Such plasticity need not challenge honesty under trade-off-based theory, but it does raise questions about the precision and resolution of the information conveyed.

In pooling equilibria, signallers in several condition states produce the same signal, creating ‘pools’ of types that receivers cannot discriminate [14,20,36,37]. When need is modelled with multiple degrees, partial pooling equilibria emerge in which only some states use the same signal. For instance, if offspring can be desperate, needy or healthy, desperate and needy individuals may rely on one signal while healthy individuals use another; parents can thus distinguish healthy from needy young, but not desperate from moderately needy ones. These equilibria are only partially informative yet remain honest because individuals in sufficiently different states send different signals, and they can be stable under at least some circumstances [18,38,39]. Hybrid equilibria provide another route to partially honest communication [40,41]. Here, pooling is probabilistic rather than fixed: one type may always produce a signal, whereas another type produces it only occasionally, and receivers may also respond only with some probability. In a parent–offspring context, for example, needy young ones may consistently beg, while less needy young ones sometimes use the same signal; parents then obtain statistical information about need, but not a reliable one-to-one mapping between signal and state. A further, non-exclusive possibility is that begging, or some of its components, did not evolve primarily to encode hunger at all. Attention-seeking display theory proposes that conspicuous signals may function mainly to attract or secure receiver attention rather than to convey detailed information about signaller quality or state [42]. For example, early begging components, such as rapid gaping or calling, might function partly to direct parental attention to a chick, whereas later or sustained components may provide coarser information about deprivation. This possibility complements, rather than replaces, pooling and hybrid-equilibrium interpretations, and further underscores the need to ask not only whether begging is honest, but also how much information about hunger its different components actually convey.

The widely accepted view that begging is highly informative rests on three main lines of suggestive evidence. First, begging correlates with food deprivation, and parents appear to adjust provisioning accurately [8,18]. Yet such correlations are equally consistent with partly honest communication [41,43]. The distinction lies in whether the information conveyed is high (separating equilibria) or low (pooling equilibria), but this has never been quantified. Second, most begging traits vary continuously [44,45] and could potentially function as graded signals conveying fine-scale information, as expected under separating equilibria [18]. Third, begging comprises multiple components—posture, calls and gaping—that are assumed to collectively increase its overall information content [7,19,27]. However, none of the latter assumptions has yet been tested empirically. Although begging is often assumed to function as an information-rich, graded signal, most studies rely on simplified metrics [43] and rarely quantify the actual information content of the display or its individual components. Questions about begging honesty may be more tractable when framed in terms of information exchange rather than strict signal–state matching [46], yet information-theoretic approaches remain largely absent from begging research (but see [18]). Further empirical work using improved methods is required to quantify the relationship between begging intensity and nestling condition [43].

Researchers typically assess honesty by regressing begging performance on nestling state [3], but conclusions based on simple regression or other univariate analyses often overlook important sources of variation. For example, failing to account for interactions with variables that also influence begging can inflate the estimated effects of key predictors, such as hunger [47]. Also, individual begging plasticity generates ‘random’ variation—manifested as differences in both intercepts and slopes of the begging–hunger relationship. While such variation is sometimes attributed to adaptive dishonesty [48], it could equally reflect non-adaptive noise [2], consistent with alternative, low-cost theoretical models of begging, including multiple pooling equilibria [37], mixed equilibria [41] and other recent approaches [24,49]. Quantifying this variation is, therefore, essential for understanding the information content of begging signals [50,51].

In this study, we revisit begging honesty using an approach that explicitly incorporates alternatives to separating equilibria, particularly partial pooling equilibria. First, we examine how begging predicts food deprivation in wild broods and in the laboratory, testing whether postural begging functions as an information-rich, graded signal by analysing its fine temporal structure—a key aspect of signal design [52]. Graded signals are expected to include multiple levels that clearly encode different, including intermediate, states [53]. Second, we assess the effectiveness of multi-component (postural and vocal) begging displays as indicators of hunger using both random regression and a more innovative approach based on information-theoretic [3] and machine learning metrics [54].

## 2. Materials and methods

**T**he study was conducted on a population of spotless starlings (*Sturnus unicolor*) breeding in nest boxes in central Spain. This species typically lays two clutches per season, with a modal brood size of four nestlings [55]. Nestlings hatch asynchronously, fledge after 21–22 days [56] and are fed by both parents [57]. The hatching date of the first egg was determined through regular nest inspections.

We observed begging behaviour when nestlings were 6 days old. At this stage, nestlings grow at peak rates [56], creating substantial variation in nutritional condition. Physical interference among nest-mates is still limited, so nestlings obtain virtually all their food through begging. To characterize begging under both natural and controlled conditions—and to analyse its fine structure without interference from nest-mates—we measured begging in the field and in a standard laboratory ‘hunger experiment’ set-up [58,59]. A key requirement for relating begging to food deprivation is establishing a protocol that keeps intake rates and inter-feeding intervals within natural limits while accounting for natural variation in parental feeding schedules [60]. Continuous recordings from 48 nests containing three to five chicks yielded a mean daily inter-feeding interval of 59.1 ± 4.27 minutes standard error (s.e.) [57]. This interval was calculated as 60/*N*, where *N* is the mean number of feedings received per chick per hour (*N* = *T*/[*B* × *t*], with *T* being the total number of feedings recorded at a nest, *B* being the brood size, and *t* being the number of observation hours). Thus, the estimate refers to the average time between successive feedings received by an individual chick, not to the interval between parental visits to the brood. However, feeding rates fluctuate markedly throughout the day; during the early-morning peak—when deprivation times are shortest—feeding rates can be up to four times higher. To examine how this variation affects begging, we recorded begging in natural broods during the morning peak (mean inter-feeding interval 19.3 ± 1.8 minutes s.e., median = 16.8 minutes; *N* = 24) and conducted the laboratory experiment under a feeding schedule matching the mean daily inter-feeding interval.

### 2.1. Observations at natural nests

We selected 24 first broods containing four or five 6-day-old chicks to examine the effect of hunger on postural begging intensity and parental feeding. Using an infrared video camera (BOBLOV PD50-32GB), we recorded parent and nestling behaviour during the early-morning feeding peak. Before recording, all nestlings were fed to satiation on demand with one to five mealworm larvae, weighed and individually marked. A trained observer, blind to the study's aims, analysed the first 3 hours of footage after the first parental visit at reduced speed (1/25 frames s^–1^). We scored 934 natural feeding events.

### 2.2. Laboratory experiment

We selected 25 first-brood nests containing two to six 5-day-old chicks. The day before the experiment, two intermediate-mass-ranked chicks from each brood were transported to the laboratory and housed in artificial nest cups under controlled temperature (*ca* 35°C) and humidity (*ca* 60%). To simulate parental behaviour and ensure habituation to laboratory conditions, nestlings were conditioned to a begging stimulus—a playback of a parental feeding call recorded from the same population—and were fed standard-sized superworms (*Zophobas morio*) on demand during at least five conditioning trials.

The next morning, we weighed the nestlings, placed them in individual artificial nests and provided four routine feeding trials at 45-minute intervals before the begging trials. Food consisted of alternating tiny omelette chunks (boiled egg) and commercially supplied insects of highly homogeneous size. Prey size was, therefore, standardized as far as possible within each food type, while ration mass and total food intake were adjusted to nestling mass (see supplementary material for rationale and full protocol). After the final feeding trial, we recorded begging behaviour in a recording chamber at 30, 60 and 90 minutes after the last meal. Each trial presented two stimuli using the same feeding-call playback, with sufficient time between stimuli for begging to extinguish. Postural begging was recorded with a digital video camera (Panasonic HC-V180), and acoustic begging was recorded with a digital audio recorder (Marantz Professional PMD620) positioned 5 cm from the nest cup. After the final trial, nestlings were fed ad libitum, blood-sampled for sex determination and returned to their original nests.

### 2.3. Measuring chick and parental behaviour

A trained observer, blind to the study's aims, analysed the videos at reduced speed. Postural begging intensity was scored using a standard five-level scale: 0 = no begging; 1 = gaping, neck flexed, body horizontal; 2 = gaping, neck extended, body horizontal; 3 = gaping, neck extended, body raised off the nest floor, back reclining; 4 = fully stretched, vertical back, sometimes with wing flapping. This scale allows comparison with most previous studies [44,61–64]. In natural broods, we recorded the timing of parental visits, identity of fed chicks, maximum postural intensity, begging rank order and two hunger proxies: deprivation time since the previous feeding and the size of the last food item (relative to the parent's bill length, measured to 0.25 units). We divided the nest cup into eight equal sectors, recorded the sector occupied by each nestling and the sector from which each parent fed, and calculated spatial profitability as the fraction of total feedings that each parent delivered to the sector occupied by a given chick, relative to the parent's position during that visit [65]. In the laboratory, we obtained continuous samples of each begging display, measuring the duration spent at each postural level. From these, we calculated total begging duration, mean postural intensity and maximum postural intensity. Mean intensity was computed by summing the duration at each level multiplied by its score and dividing by total begging time (e.g. [66,67]). We also recorded the latency from the onset of each stimulus to the start of begging.

We analysed begging vocalizations using Avisoft SAS-Lab Pro v. 5.1.23 [68]. Begging calls consisted of multi-band trills with varying degrees of frequency modulation (supplementary material, figure S1). From sound spectrograms (256 fast Fourier transform (FFT), 162 Hz bandwidth, 11.6 ms time, 43 Hz frequency resolution), we extracted temporal and spectral measurements. Total vocal bout duration, number of calls and latency to call after the playback stimulus were recorded manually. For automatic measurements, we selected a sample of the first calls following each playback (up to 10 per stimulus) and quantified call duration, peak frequency, maximum and minimum frequency and call entropy. Because spectral measurements in multi-band, frequency-modulated calls can vary markedly across adjacent time segments, each frequency variable was measured at four points: two around the point of maximum amplitude and two across the entire call element (supplementary material). For whole-element measures, robust estimates of low and high frequencies were obtained using the 25th (P25) and 75th (P75) frequency percentiles rather than instantaneous extrema. Frequency modulation was quantified as the s.d. of these two frequency percentiles within each call. Single spectral measures—peak frequency, maximum frequency, minimum frequency and entropy—were then derived as linear combinations of the multiple measurements using principal components analysis (supplementary material, table S1). In total, 1904 calls were analysed.

### 2.4. Statistical analysis

#### 2.4.1. Modelling food deprivation as a predictor of begging performance

We conducted all statistical analyses in R 4.1.3 [69]. To improve model stability, convergence and the accuracy of parameter estimates [70], all independent variables were *Z-*transformed (mean-centred and scaled to 1 s.d.). This transformation also places variances on a comparable phenotypic scale, improving the estimation of variance components [71].

To determine how begging components predicted food deprivation, we analysed the data using mixed-effects models and adopted a top-down model selection strategy [72]. For each response variable, we first identified an appropriate random-effects structure (supplementary material, table S2) and subsequently selected the fixed-effects structure, comparing alternative nested models using either information-theoretic criteria or likelihood-ratio tests, depending on model type and estimation method (supplementary material). For analyses of postural intensity in natural broods, we included chick and parental visit as crossed random intercepts (both nested within nest). Including parental visit accounts for variation in parental behaviour (e.g. position) and for chick–chick interactions during the same feeding bout. For analyses of begging order, we included the chick nested within the nest as the random intercept. Time since last feeding, prey size and nestling mass were included as candidate random slopes. For models fitting parental response, random effects included nest as a random intercept and random slopes for nestling behavioural traits and spatial profitability (at the level of the parent within nest; supplementary material, table S3). For laboratory analyses, the chick nested within the nest was included as a random intercept, with time of food deprivation as a random slope (supplementary material, table S4).

In natural broods, we used ordinal mixed models [73] for begging order and maximum postural intensity and linear mixed models [74] for the remaining nestling response variables. Begging order ranks were standardized across brood sizes following Smith & Montgomerie [75] (see supplementary material for further details). Main predictors included time since the last feeding and prey size. Brood-mean-centred nestling mass, hatching date and brood size, and their interactions with the main predictors were included as fixed effects in all models [49,76]. For parental responses, we fitted generalized linear mixed models with a binomial error distribution, using whether a nestling was fed during a parental visit as the response variable. Predictors included nestling behaviour (maximum postural intensity and begging order), relative nestling mass, spatial profitability and the same covariates and two-way interactions described above. Cases in which nestlings did not beg were excluded from all analyses.

In the laboratory experiment, we first analysed several whole-display response variables—total begging duration, latency to beg, time displayed at maximum intensity and mean and maximum postural intensities. We then examined the fine structure of each display by measuring the time spent in each postural level and the probability of reaching each level with different visual components. Predictors included time since last feeding, nestling sex, body mass, brood size in the natal nest, date and stimulus order (1 or 2). Interactions between food deprivation and all predictors except stimulus order were considered. Single-measure acoustic variables, including calling rate, total vocal output duration, latency to vocalize, distance to maximum amplitude, call duration and the s.d. of frequencies P25 and P75, were analysed using the same modelling framework. To combine the multiple measures of peak frequency, maximum frequency (including the frequency P75), minimum frequency (including the frequency P25) and entropy, we conducted separate principal components analyses and used the resulting first principal component (PC1) scores as response variables (supplementary material, table S1). For models analysing individual call characteristics, we included the sequential order of calls within each stimulus as a fixed effect, as acoustic structure can vary with call sequence [50,77,78]. Further details are provided in the supplementary material.

To account for multiple perspectives and response variables when testing whether hunger predicts begging behaviour, we applied Benjamini–Hochberg corrections to control the false discovery rate. Additional methodological details are provided in the supplementary material .

#### 2.4.2. Effectiveness of multi-component begging signals

We quantified the effectiveness of individual begging components using two complementary approaches.

First, we used coefficients of determination (*R*²) to estimate the proportion of variance in each begging component explained by food deprivation [3]. *R*² can be partitioned into variance explained by individual predictors using semi-partial coefficients of determination ((part)R²) computed with the partR2 package [79]. Semi-partial *R*² is suitable for continuous variables and can isolate the variance explained by a predictor while accounting for its interactions, providing more reliable estimates [79]. However, it cannot be computed when the predictor of interest is specified as a random slope, so all (part)R² values reported refer to models without random slopes. To compare how strongly food deprivation explained variation across different begging components, we also computed estimates of the total variance explained by all fixed effects combined (marginal *R*^2^) and that explained by both fixed and random effects (conditional *R*²) following Nakagawa & Schielzeth [80]. Marginal and conditional *R*² values were obtained using r.squaredGLMM() in the MuMIn package [81], which is valid for models containing random slopes [82]. Ratios of semi-partial *R*² to conditional *R*² were computed to identify begging components for which semi-partial *R*² estimates might be inflated owing to the presence of random slopes. For ordinal models, we calculated semi-partial coefficients as the difference in marginal *R*² between models with and without the predictor of interest.

Second, we quantified mutual information (MI), which measures the reduction in uncertainty about nestling food deprivation achieved by a receiver after observing a given begging signal level [3,83]. We calculated MI using the mutinformation() function in the infotheo R package [84]. MI is scale-independent and makes no distributional or regression assumptions, but it requires categorical inputs; continuous variables must therefore be discretized, and MI estimates depend on the number and type of categories used. We applied two complementary discretization approaches. First, we used rank-based binning (equal-frequency bins) with dplyr::ntile [85], which yields similarly sized categories and therefore stable estimates even for skewed distributions. Second, we used equal-width binning (equal intervals on the measurement scale) with infotheo::discretize (method = ‘equal-width’) [84], which preserves absolute differences in deprivation time or signal magnitude and is directly interpretable in physical units (e.g. seconds and Hz). The two approaches are expected to diverge for strongly skewed variables: equal-width binning can produce sparse or empty tail categories and lower estimated information because most observations cluster in a few bins, whereas rank-based binning maintains populated categories and typically gives higher, more stable estimates. When applied to deprivation time, equal-width binning corresponds to absolute deprivation magnitude, whereas rank-based binning corresponds to relative need (percentile rank). When applied to a signal component, the same contrast reflects alternative assumptions about receiver perception rules: fixed thresholds on the measured scale (equal-width) versus sensitivity to relative levels of expression (rank-based). Unless stated otherwise, continuous begging variables were initially discretized into percentiles to minimize loss of variation.

Some acoustic begging variables (e.g. frequency or entropy) were measured at the level of individual calls within a begging bout (stimulus), whereas hunger state and postural displays were constant across calls within a bout. Treating calls as independent observations would, therefore, result in pseudoreplication, because multiple calls from the same bout do not represent independent signalling events. Conversely, collapsing data to a single bout-level summary (e.g. averages) would discard potentially meaningful variation among calls and is difficult to justify, given that parents often make allocation decisions early in a bout, before it is completed. We therefore retained call-level acoustic data but controlled explicitly for pseudoreplication by computing bout-weighted (1/*n,* where *n* = number of calls in a bout) probabilities in MI analyses, ensuring that each bout contributed equally regardless of the number of calls it contained.

MI is expressed in bits or nats, which can be difficult to interpret biologically; hence, we report results in terms of uncertainty reduction, calculated as the fraction of the initial uncertainty about deprivation that is resolved, on average, after observing a begging component. This standardizes MI relative to the maximum possible uncertainty in the system, enabling direct comparisons across begging components and signalling contexts (e.g. laboratory and field). Uncertainty reduction was computed as MI divided by the entropy of deprivation time.

Finally, we quantified how the effectiveness of the composite begging signal changes as the display unfolds and receivers can integrate progressively more components (e.g. latency, posture and vocalizations), using begging bouts recorded under laboratory conditions. To obtain a receiver-centred measure of signal effectiveness, we evaluated how accurately deprivation state could be inferred from the multi-component display using a random-forest classifier with *K*-fold cross-validation (*K* = 5) implemented in the randomForest package [86]. Predictors were entered sequentially according to their temporal availability within a bout, mirroring the order in which parents could plausibly acquire information: (i) front-line components available at display onset (latency followed by maximum postural intensity), (ii) mid-bout components that can be assessed during the ongoing display (duration at maximum intensity, calling rate, and temporal and spectral acoustic properties of calls), and (iii) end-point components that can only be evaluated after bout completion (total bout duration and mean postural intensity). Within the latter two temporal subsets, components were ordered by their individual effectiveness, and to assess robustness, we repeated the analysis using alternative orderings derived from semi-partial *R*^2^ and from MI computed under the two discretization methods. For each subset size, we estimated out-of-sample accuracy relative to a baseline (majority-class) decoder. Because postural and vocal measures of latency and total duration were highly correlated, we retained the vocal measures, which offer higher temporal precision; substituting the postural counterparts did not change the results. To avoid pseudoreplication, cross-validation was grouped by begging bout (defined as the unique chick × deprivation × stimulus combination), ensuring that all calls from a given bout were assigned to the same training or test fold. In addition, calls were weighted by 1/*n* (where *n* is the number of calls in the bout) so that each bout contributed equally to the overall accuracy estimate, regardless of the number of calls sampled.

#### 2.4.3. Signal effectiveness and overlap in pooling coding schemes

To explicitly adopt a partial pooling-equilibria perspective on begging signals, we assessed how distinctly different postural levels (as perceived by a human observer) mapped onto underlying hunger, measured as deprivation time.

First, we quantified the degree of overlap between the deprivation times associated with each pair of postural-intensity levels. For each signal level, we characterized the empirical distribution of deprivation times and then measured how much these distributions overlapped when compared pairwise (supplementary material, figure S2). This overlap coefficient ranges from zero (no overlap, fully distinct signals) to one (complete overlap, no discriminative value), providing an intuitive measure of how well different postural displays separate individuals in different need states. A more detailed description of the statistical procedure is provided in the supplementary material.

Second, we evaluated how the signal effectiveness of the postural display, considered as a discrete signal, changed with the number of postural level categories (pools) considered. We analysed four separate postural levels, three levels (grouping 3+4) and two pooled categories (grouping 1+2 and 3+4), and each scheme was examined with and without a ‘non-begging’ category. To assess the robustness of our MI-based conclusions, we computed three additional measures of discrete signal effectiveness to quantify how accurately begging intensity reflects deprivation state (consistency) and how well a receiver can infer that state (reliability and Bayes decoding accuracy) across coding schemes with different numbers of signal pools.

— Average consistency quantifies how regularly a given output (postural level) is produced when a particular input (deprivation level) is true, ranging from 0 to 1. It identifies the most likely output for each input and sums these likelihoods weighted by the prior probability of each input [3].
— Reliability measures the probability that a deprivation state is correctly inferred after receiving a signal, updating prior expectations via Bayes' theorem. Average reliability weights the reliability for each state by its prior probability and sums these values to yield an overall measure of receiver performance for a given coding scheme [3]. Computing reliability requires an *a priori* mapping between each signal level and a specific deprivation category. As no such mapping existed for our system, we assumed only that signal and condition categories were ordinally aligned (i.e. higher postural levels correspond to longer deprivation times).
— Bayes decoding efficiency (normalized Bayes decoding accuracy), a standard measure of optimal classification performance that estimates how well an ideal receiver could classify deprivation states based solely on the empirical frequency distributions of signals, equivalent to applying the optimal decision rule in statistical pattern recognition: selecting the state with the highest posterior probability given the signal [87,88] (see supplementary material for further details). This metric does not require any prior assignment or ordering of signal or condition categories. In addition, it is well-suited to heterogeneous or highly overlapping coding schemes.

In natural broods, deprivation time varied continuously, so we discretized it in two complementary ways. First, we explored multiple bin sizes (4, 8, 12 and 16, each with both discretization methods) to assess how different levels of resolution in deprivation time influenced estimates of signal effectiveness. Second, we defined three biologically motivated deprivation categories consistent with a partly informative (pooling-equilibria) framework, in which parents respond to broad differences in chick hunger rather than to precise, fine-scale variation in deprivation time. When delivering a single prey item per visit, parents probably ignore the exact time since each chick last fed, but would benefit from identifying the most and least deprived offspring before allocating food. Simple expectations from brood-feeding dynamics suggest that the hungriest chick typically experiences deprivation intervals about 1.5 times the brood average, whereas the least hungry chick experiences intervals roughly half as long. Using the mean deprivation time in our sample as a reference, we therefore classified observations into short, medium and long deprivation categories that matched the 30-, 60- and 90-minute treatments used in the laboratory experiment. Full details of the rationale and calculations are provided in the supplementary material.

## 3. Results

### 3.1. Observations at natural broods

During the morning peak of feedings, a focal nestling received food every 16.56 minutes on average (± 1.78 s.e., *N* = 112 chicks), with inter-feeding intervals ranging from 3 s to 3.2 hours. Time since the last feeding, interacting with previous meal size, significantly predicted begging order within broods (table 1). The negative relationship between begging order and deprivation time was steeper when nestlings had consumed a large prey item in the previous meal (supplementary material, figure S3). Nestlings also increased their maximum postural intensity as deprivation time increased and when their previous meal was small. For similar levels of deprivation and prey size, smaller nestlings and those in larger broods attained higher postural intensities (table 1).

**Table 1.** Parameter estimates (± s.e.), *z-*values and *p-*values from ordinal mixed models explaining maximum postural intensity and begging order in natural broods.

	estimate	s.e.	*z*	*p*	corrected *p*^1^
**maximum postural intensity**					
deprivation time	0.72	0.09	7.96	<0.001	<0.001
brood size	0.43	0.17	2.62	0.009	
nestling mass	–0.20	0.07	–3.01	0.003	
prey size	–0.15	0.07	–2.12	0.034	0.034
**begging order**					
deprivation time	–0.44	0.07	–6.67	<0.001	<0.001
prey size	0.20	0.05	3.99	<0.001	
deprivation time x prey size	–0.14	0.06	–2.21	0.027	0.034

^1^ Benjamini–Hochberg-corrected *p-*values for the effects of food-deprivation time and prey size

Deprivation time explained substantially more variance than prey size for both begging order (5.6% versus 0.8%) and maximum postural intensity (7.8% versus 0.5%). It also accounted for most of the variance explained by all fixed effects combined (77.8% for begging order and 64.5% for postural intensity; table 2). Nonetheless, both components of the visual begging display contained only modest information about nestling hunger. The reduction in uncertainty about deprivation time available to a parent observing each component of the begging display was 0.4–0.7% for begging order and 2.4–2.6% for postural intensity, depending on discretization method, increasing to 3.3–4.1% when both components were considered jointly. Information relating to prey size was roughly half as large (table 2) and 2% for the two components combined. As shown in figure 1, deprivation times overlapped substantially across postural intensity levels. On average, overlap between maximum postural levels was 75.9% (± 0.07 s.d.), ranging from 64.1% (levels 1–4) to 87.7% (levels 3–4).

**Table 2.** Variance explained by deprivation time and prey size in postural begging components in natural broods and corresponding MI. Shown are semi-partial *R*² (isolated predictor effects), marginal *R*² (fixed effects) and conditional *R*² (fixed + random effects) from ordinal mixed models. MI is reported in bits and as percentage uncertainty reduction (%H) under two discretization schemes for deprivation time (rank-based and absolute-scale).

				mutual information MI			
	**coefficient of determination *R*^2^**			**bits**		**%H**	
	**semi-partial**	**marginal**	**conditional**	**ranks**	**absolute**	**ranks**	**absolute**
**maximum postural intensity**		0.121	0.541				
deprivation time	0.078			0.158	0.122	2.38	2.61
prey size	0.005			0.044		1.43	
**begging order**		0.072	0.083				
deprivation time	0.056			0.044	0.017	0.67	0.37
prey size	0.008			0.011		0.37	
deprivation time x prey size	<0.01

**Figure 1.**
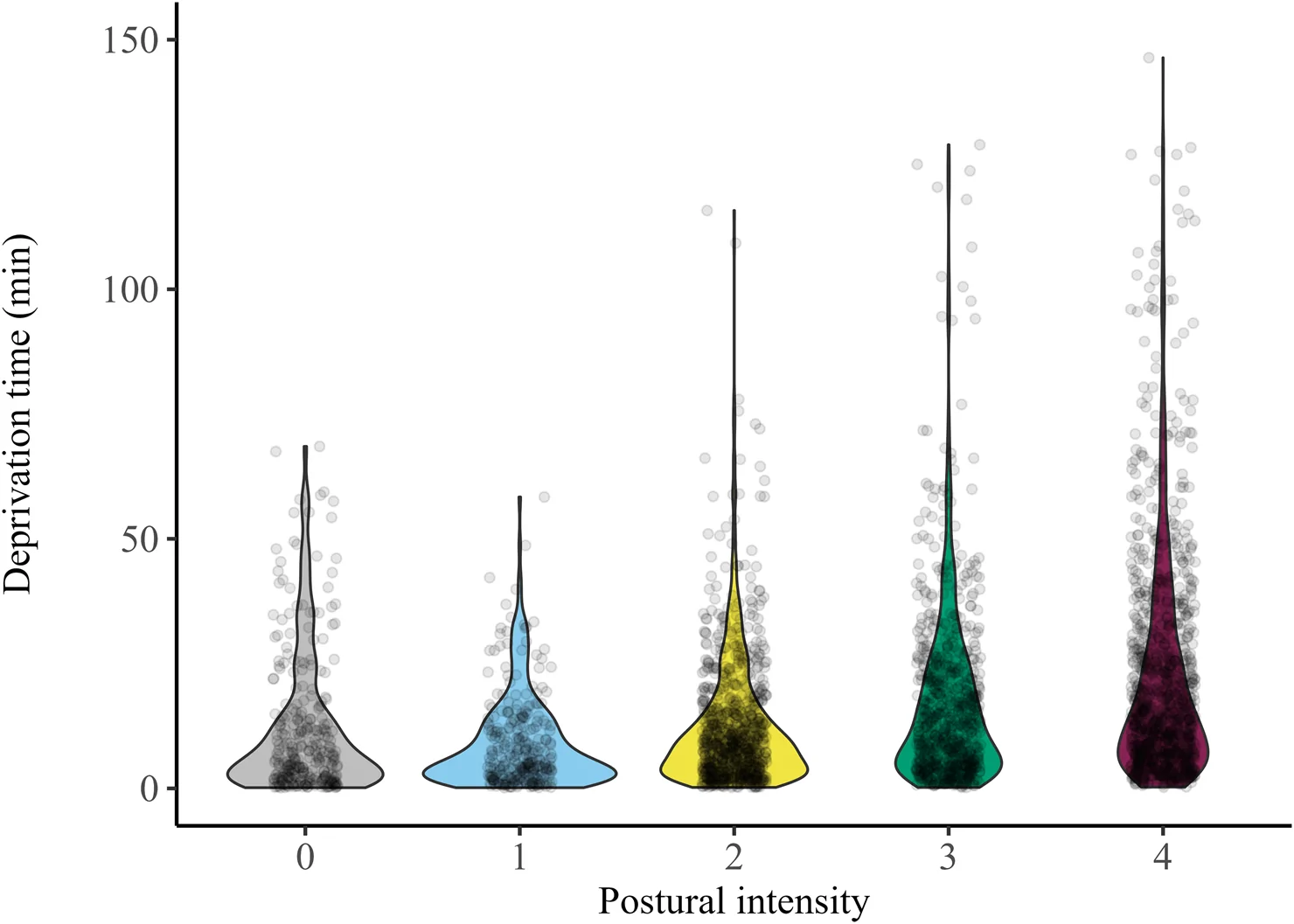
Deprivation time since the last feeding for all observations in natural broods, plotted by maximum postural intensity (0 = no begging). Three extreme values (level 4: 167.3 minutes; level 3: 169.9 and 169.2 minutes) are omitted to improve the y-axis scale.

Parents delivered a single feeding in 93.5% (± 1.23 s.e., *N* = 24 broods) of observed visits. Adults took on average 3.81 s (± 6.89 s.e., *N* = 24 broods; 75% = 4.0 s) to transfer food after entering the nest box, but remained inside much longer (16.1 s ± 27.16 s.e., IQR = 12-17 s, *N* = 24; excluding visits involving brooding or cleaning). Parental allocation decisions were complex, integrating several nestling traits alongside begging order and postural intensity. Parents preferred chicks that begged first, regardless of their postural intensity, but postural intensity increased the probability of being fed for chicks that begged later (table 3; supplementary material, figure S4). In addition, heavier nestlings and those positioned in more favourable nest locations were more likely to receive food (table 3).

**Table 3.** Parameter estimates (± s.e.), *z*-values and *p-*values from a logistic mixed model explaining the probability of parental feeding in natural broods.

	estimate	s.e.	*z*	*p*
intercept	–1.28	0.07	–17.3	<0.001
postural intensity	0.61	0.07	8.22	<0.001
begging order	–0.58	0.07	–7.90	<0.001
spatial profitability	0.43	0.09	4.82	<0.001
postural intensity × begging order	0.33	0.07	4.50	<0.001
body mass	0.29	0.05	5.36	<0.001
brood size	–0.21	0.06	–3.55	<0.001

### 3.2. Laboratory experiment

#### 3.2.1. Postural begging

All nestlings in the laboratory begged when stimulated. Begging displays lasted on average 13.1 s (± 1.25 s.e., *N* = 298). Regardless of the maximum intensity reached, almost all displays included lower postural levels (99% of displays with maximum level 4, 98% with level 3 and 93% with level 2). Level 2 comprised the largest proportion of total display time (49.1% ± 1.68 s.e., *N* = 274), followed by level 3 (42.9% ± 1.84 s.e., *N* = 140), level 1 (31.2% ± 1.22 s.e., *N* = 268) and level 4 (19.1% ± 1.44 s.e., *N* = 103). To examine how this variation related to food deprivation, we proceeded in two steps. First, we characterized the fine temporal structure of the display. Second, we tested how deprivation time affected the postural display both as a whole and when decomposed into its components.

Nestlings typically reached their maximum postural intensity at the very start of the display, after which intensity declined progressively. As shown in figure 2, submaximal levels tended to appear later in the sequence, with lower levels occurring progressively later, indicating that these levels largely reflect nestlings returning to a resting posture after initially reaching maximum intensity. Displays that reached higher maximum intensities were also longer overall (estimate ± s.e. = 0.782 ± 0.317, *t*₁_,_₄₆ = 2.466, *p* = 0.018). However, the duration of the level at which maximum intensity was attained was longer for lower-intensity maxima (9.90 ± 1.76 s s.e. for level 1 and 8.39 ± 0.26 s s.e.E for level 2) than for higher-intensity maxima (4.10 ± 0.57 s s.e. for level 3 and 2.61 ± 0.19 s s.e. for level 4) (figure 2).

**Figure 2.**
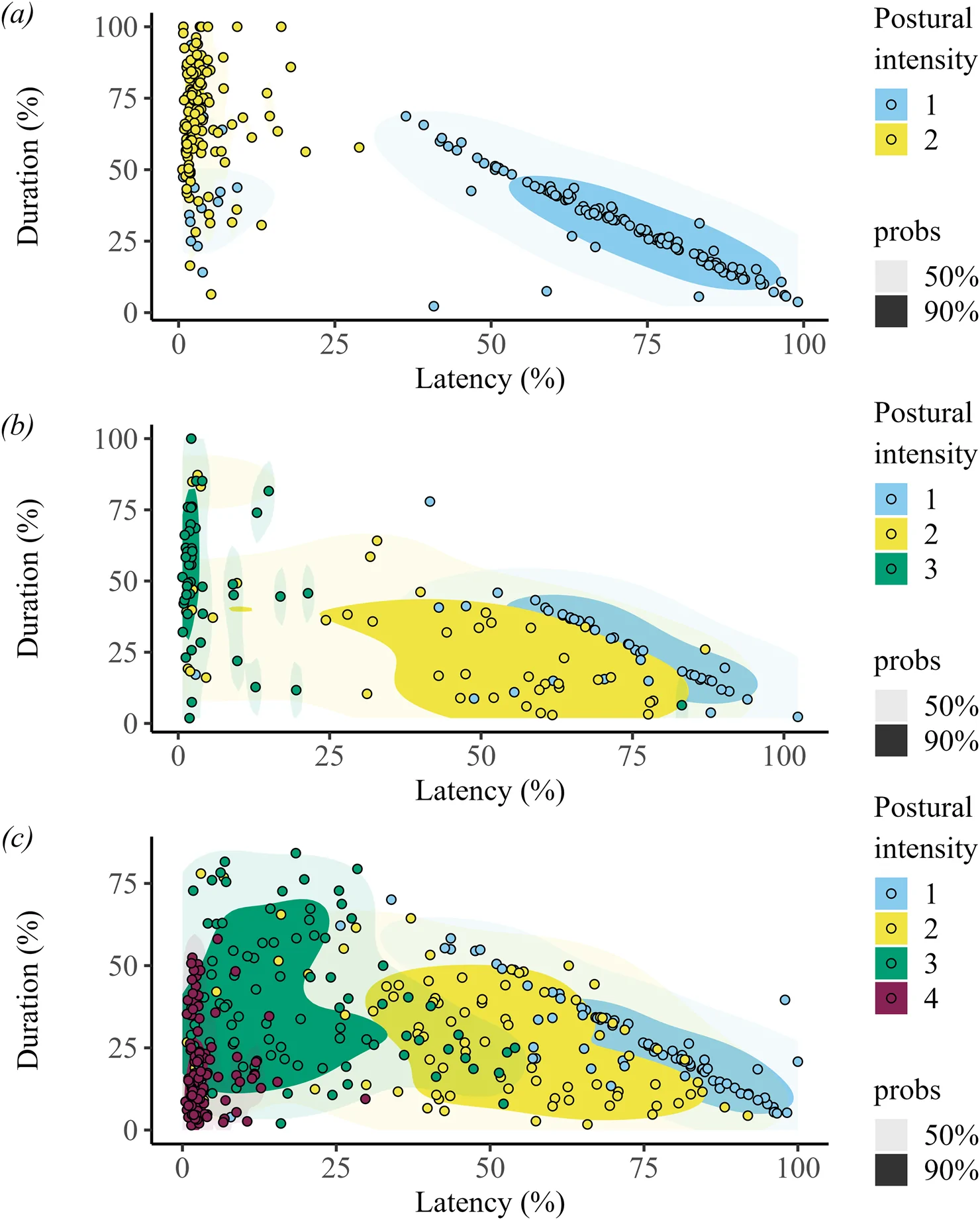
Fine temporal structure of visual begging displays reaching different maximum postural intensities. Shown are the central 50 and 90% distributions of time spent at each postural level (y-axis) as a function of latency to first reach that level (x-axis), both expressed as percentages of total display duration. Panels represent displays with maximum intensity (*a*) level 2, (*b*) level 3 and (*c*) level 4.

Hungrier nestlings displayed for longer, and this effect interacted with brood size: nestlings coming from larger natal broods produced longer displays regardless of deprivation time (supplementary material, figure S5). Deprivation time did not significantly affect latency to beg, neither when both stimuli were combined (table 4), nor when considering latency to beg after the first stimulus only (estimate *β* = –0.003 ± 0.006, *t*_21_ = –0.458, *p* = 0.651). Both maximum and mean postural intensity increased after a longer deprivation time (table 4). Nestlings also reached lower maximum intensities on their second stimulation. Time spent displaying at the maximum postural intensity (irrespective of the level attained) tended to decrease with increasing deprivation time (figure 2), but this effect was not statistically significant (table 4).

**Table 4.** Parameter estimates (± s.e.) for models testing the effect of deprivation time on overall postural begging performance in laboratory nestlings. Shown are *t-*values for mean postural intensity, total display duration and latency to beg and *z-*values for maximum postural intensity. Uncorrected and Benjamini–Hochberg-corrected *p-*values are reported for deprivation time. Sex is included as a categorical factor (male reference level).

	estimate^1^	s.e.	*z* or *t*	*p*	corrected *p*^2^
**mean postural intensity**					
intercept	2.04	0.07	31.1	<0.001	
deprivation time	0.15	0.03	5.01	<0.001	<0.001
**maximum postural intensity**					
deprivation time	1.00	0.21	4.08	<0.001	<0.001
stimulus	–0.43	0.14	–3.08	0.002	
**duration of postural display**					
intercept	13.0	0.49	26.5	<0.001	
body mass	1.06	0.44	2.43	0.019	
brood size	0.63	0.49	1.28	0.216	
deprivation time × brood size	–0.50	0.20	–2.49	0.014	0.022
deprivation time	0.40	0.20	2.02	0.045	
**latency to gape**					
intercept	0.21	<0.01	53.0	<0.001	
deprivation time	–0.01	<0.01	–1.58	0.126	0.126
**time at maximum intensity**					
intercept	6.22	0.41	14.7	<0.001	
deprivation time	–0.40	0.20	–1.95	0.053	0.065
sex^3^	–0.12	0.51	–0.24	0.813	

^1^ Predictors other than deprivation time (sex, body mass, brood size, date and stimulus) and their interactions that did not improve model fit are not shown.

^2^ Benjamini–Hochberg-corrected *p*-values for the effect of food-deprivation time.

^3^ Reference level: male.

Next, we analysed how food deprivation affected the degree of postural stretching, i.e. the likelihood of reaching each postural intensity level. Most displays included levels 1 and 2 at some point in the sequence—whether as maximum or submaximal postures—regardless of deprivation time (88–92 and 90–91%, respectively). The proportion of displays including level 3 increased with deprivation (34% at 30 minutes, 48% at 60 minutes and 61% at 90 minutes), and the same was true for level 4 (19, 36 and 48%, respectively). However, when we considered only the fraction of displays in which a level was reached as the maximum, only levels 2 and 4 differed significantly between the two extreme hunger levels (30 versus 90 minutes) ( figure 3; Fisher's exact test: *p* < 0.001 for levels 2 and 4, *p* = 0.854 for level 3). Thus, the increased occurrence of level 3 with hunger reflected its appearance as a submaximal, transitional posture after nestlings had already reached level 4 at the display onset (figure 2). Hungrier nestlings were more likely to reach level 4 and less likely to reach levels 2 and 1 ( table 5). Chicks in the laboratory rarely reached level 1 as their maximum intensity (eight displays, 2.7%), and only after 30 or 60 minutes of deprivation (five and three displays, respectively). Nestlings were less likely to attain level 4 on the second stimulus than on the first ( table 5).

**Figure 3.**
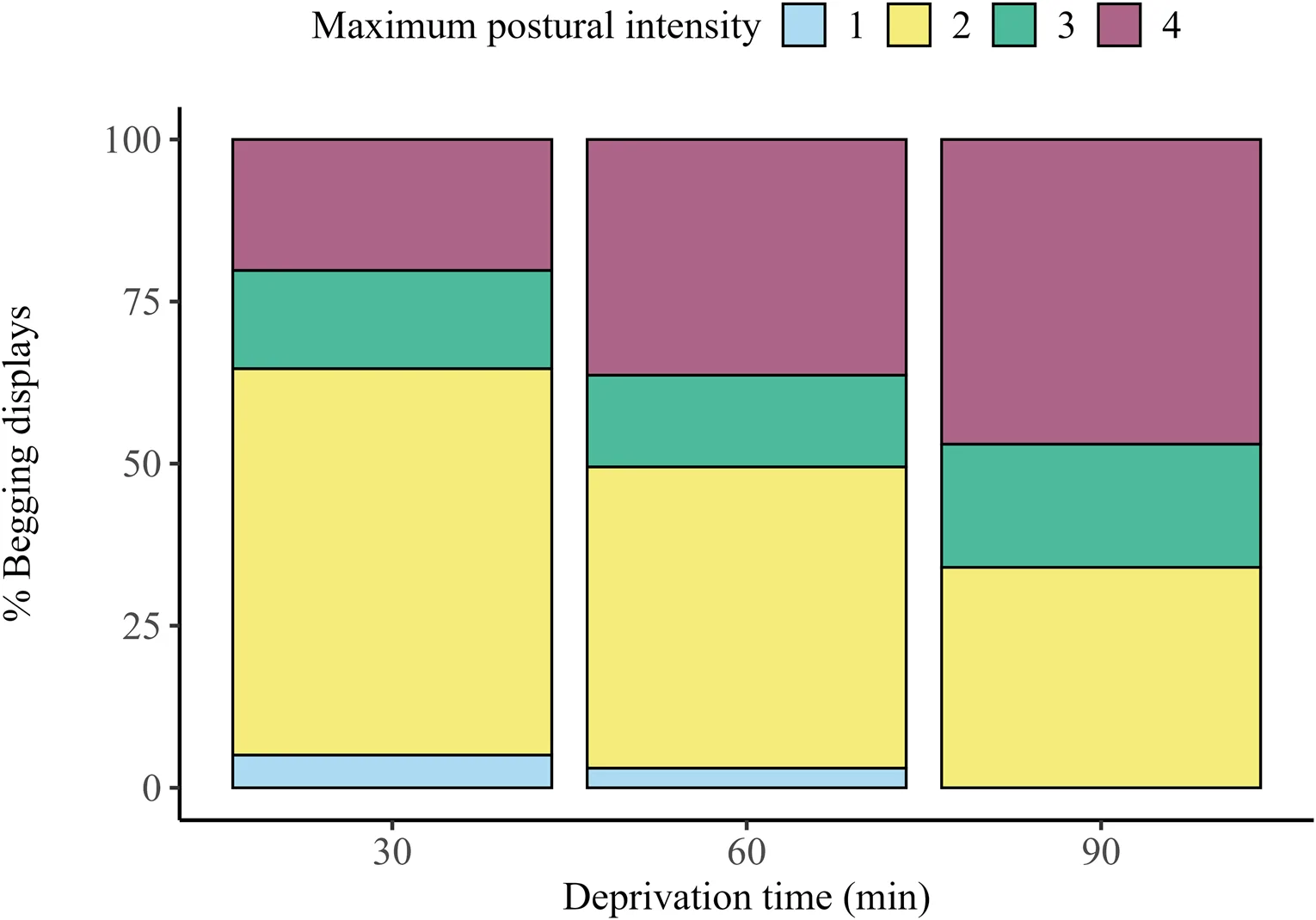
Proportion of laboratory nestling displays reaching each maximum postural intensity level as a function of deprivation time.

**Table 5.** Parameter estimates (± s.e.) for models analysing the probability and duration of decomposed postural begging levels in laboratory nestlings. Shown are *z-*values for the probability of reaching a given level as the maximum and *t-*values for time spent at maximum intensity and at each postural level. Duration results are presented for all displays and for displays in which a given level was reached as the maximum.

	estimate^1^	s.e.	*z* or *t*	*p*	corrected *p*^2^
**probability of reaching level 4**					
intercept	–1.15	0.36	–3.18	0.001	
deprivation time	0.71	0.30	2.40	0.017	0.026
stimulus	–0.66	0.18	–3.63	<0.001	
**probability of reaching level 3**					
intercept	–2.05	0.34	–5.96	<0.001	
deprivation time	0.07	0.17	0.41	0.679	0.678
stimulus	0.49	0.18	2.75	0.006	
**probability of reaching level 2**					
intercept	–0.35	0.37	–0.94	0.349	
deprivation time	–0.88	0.30	–2.92	0.003	0.009
**probability of reaching level 1**					
intercept	–10.4	<0.01	–2811	<0.001	
deprivation time	–1.06	<0.01	–287	<0.001	<0.001
stimulus	1.43	0.61	2.35	0.019	
**time displaying at level 4**					
intercept	0.39	0.06	6.18	<0.001	
deprivation time	0.18	0.04	4.23	<0.001	0.001
stimulus	–0.11	0.02	–4.68	<0.001	
**time displaying at level 4 as maximum**					
intercept	2.23	0.22	10.0	<0.001	
deprivation time	0.71	0.17	4.08	<0.001	<0.001
body mass	–0.69	0.23	–3.08	0.006	
**time displaying at level 3**					
itercept	0.84	0.11	7.38	<0.001	
deprivation time	0.18	0.06	3.11	0.005	0.009
**time displaying at level 3 as maximum**					
intercept	6.56	0.49	13.5	<0.001	
deprivation time	0.17	0.41	0.42	0.677	0.679
**time displaying at level 2**					
intercept	5.70	0.35	16.1	<0.001	
body mass	0.72	0.34	2.12	0.040	
deprivation time	–0.16	0.26	–0.61	0.547	0.679
**time displaying at level 2 as maximum**					
intercept	8.81	0.43	20.7	<0.001	
deprivation time	0.52	0.23	2.23	0.027	0.038
date	0.75	0.42	1.79	0.088	
**time displaying at level 1**					
intercept	1.34	0.08	17.2	<0.001	
deprivation time	–0.06	0.05	–1.22	0.234	0.312

^1^ Predictors other than deprivation time (sex, body mass, brood size, date and stimulus) and their interactions that did not improve model fit are not shown.

^2^ Benjamini–Hochberg-corrected *p*-values for the effect of food-deprivation time.

Finally, we tested whether food deprivation could be predicted from the duration spent displaying each postural level, rather than merely from whether a given level was reached. We modelled deprivation time as either a continuous (numeric) or a categorical predictor; the latter allowed us to test for nonlinear relationships, including whether intermediate postural levels corresponded to intermediate deprivation times. When all displays were included, longer deprivation was associated with longer durations at levels 4 and 3. By contrast, deprivation time had no significant effect on the duration spent at levels 1 or 2, whether modelled as a continuous or categorical predictor ( table 5; supplementary material, table S5). Thus, time spent at levels 1 and 2 did not encode low or intermediate levels of hunger, respectively. These analyses combined cases where nestlings never displayed a level (duration = 0 s) with cases where the level appeared only late in the sequence (i.e. not as the maximum). Consequently, they confound the probability of begging with the duration of begging at that level, and they mix time spent at the initial maximum with time spent in subsequent, residual postures. To disentangle these effects, we conducted a separate analysis restricted to displays that reached a given level as the maximum (and, for level 4, excluding displays that never reached it). Within this subset, deprivation time still had a significant positive effect on the duration of level 4 (table 5). By contrast, time spent at level 3 showed no significant relationship with deprivation, whether modelled as a continuous (table 5) or categorical predictor (supplementary material, table S5). Time spent at level 2 increased with deprivation when modelled continuously (table 5) but did not vary when modelled categorically (supplementary material, table S5).

All models required random slopes except those predicting total display duration, the probability of reaching level 3, and the time spent at levels 4, 3 and 2 when these levels were attained as the maximum (supplementary material, table S4). When random slopes were removed, latency to gape became statistically significant (Benjamini–Hochberg-corrected *p* = 0.035). In addition, deprivation time entered significant interactions with other predictors for three response variables: with nestling mass for the probability of reaching level 2, with nestling sex for the time spent at level 4 (considering all displays), and with brood size and hatching date for the probability of reaching level 4 (supplementary material, table S6).

#### 3.2.2. Acoustic begging

Nestlings vocalized throughout most of the begging display, with vocal bouts lasting on average 11.4 s (± 0.20 s.e., *N* = 275). Visual and vocal durations were strongly interrelated (estimate ± s.e. = 3.29 ± 0.34, *t*₁_,_₄₀ = 9.69, *p* < 0.001), but none of the remaining acoustic variables was correlated with either maximum postural intensity or postural duration. After correcting *p-*values, three acoustic variables were significantly influenced by deprivation time: hungrier nestlings produced longer vocal bouts (again showing an interaction with brood size, as in postural duration), emitted higher-pitched calls (minimum frequency PC1), and showed reduced frequency modulation (s.d. of the frequency P75) (table 6). Variation in calling rate, acoustic latency to beg (either considering the first or both stimuli), call duration, distance from start to peak, peak and maximum frequency PC1, call entropy PC1, and the s.d. of the frequency P25 were not significantly affected by deprivation time (table 6).

**Table 6.** Parameter estimates (± s.e.) and *t*-values from models explaining variation in acoustic begging variables in laboratory nestlings.

	estimate^1^	s.e.	*t*	*p*	corrected *p*^2^
**calling rate**					
intercept	1.58	0.04	38.4	<0.001	
deprivation time	0.01	0.01	1.87	0.063	0.138
**duration of vocal bout**					
intercept	10.9	0.55	19.8	<0.001	
sex^3^	1.29	0.71	1.82	0.078	
deprivation time	0.41	0.14	2.85	0.005	0.042
stimulus	–0.37	0.14	–2.55	0.011	
deprivation time × brood size	–0.36	0.14	–2.55	0.011	
brood size	–0.23	0.45	–0.51	0.616	
**latency to call**					
intercept	0.19	<0.01	47.2	<0.001	
deprivation time	<–0.01	<0.01	–0.87	0.391	0.430
**call duration**					
intercept	0.35	0.01	27.0	<0.001	
sequence	–0.02	<0.01	–13.2	<0.001	
stimulus	–0.01	<0.01	–4.95	<0.001	
deprivation time	0.01	<0.01	1.54	0.139	0.218
**distance from start to peak**					
intercept	0.07	<0.01	15.7	<0.001	
sequence	–0.01	<0.01	–5.10	<0.001	
deprivation time	<0.01	<0.01	1.22	0.233	0.320
**peak frequency PC1**					
intercept	–0.09	0.24	–0.36	0.726	
deprivation time	0.14	0.05	2.60	0.017	0.080
**minimum frequency PC1**					
intercept	–0.06	0.24	–0.26	0.795	
deprivation time	0.16	0.05	2.90	0.008	0.042
sequence	–0.08	0.02	–4.18	<0.001	
**maximum frequency PC1**					
intercept	0.04	0.27	1.37	0.179	
sex^3^	–0.75	0.40	–1.87	0.069	
deprivation time	–0.06	0.06	–1.04	0.309	0.378
**call entropy PC1**					
intercept	–0.42	0.30	–1.38	0.177	
sex	0.98	0.37	2.62	0.013	
deprivation time	0.01	0.05	0.26	0.801	0.801
**s.d. of the frequency P25**					
intercept	0.09	<0.01	27.7	<0.001	
sequence	<0.01	<0.01	4.96	<0.001	
deprivation time	<–0.01	<0.01	–1.67	0.109	0.200
**s.d. of the frequency P75**					
intercept	0.09	<0.01	22.8	<0.001	
deprivation time	<–0.01	<0.01	–3.26	0.004	0.039

^1^ Predictors other than deprivation time (sex, body mass, brood size, date and stimulus) and their interactions that did not improve model fit are not shown.

^2^ Benjamini–Hochberg-corrected *p*-values for the effect of food-deprivation time.

^3^ Reference level: male.

With the exception of models for vocal-bout duration and calling rate, all acoustic models required random slopes (supplementary material, table S4). When random slopes were omitted, deprivation time significantly affected six of the eleven acoustic variables, with an additional marginal effect on the time from call onset to energy peak, and an interaction with brood size for peak frequency PC1 (supplementary material, table S6).

#### 3.2.3. Effectiveness of multi-component signals

Maximum postural intensity was the most effective component of the postural display in terms of its contribution to total explained variance. It was followed by the time spent at, and the probability of reaching, postural level 4, as well as by mean postural intensity; together with latency to gape, these variables showed the highest semi-partial coefficients of determination and the largest contributions to total explained variance (figure 4).

**Figure 4.**
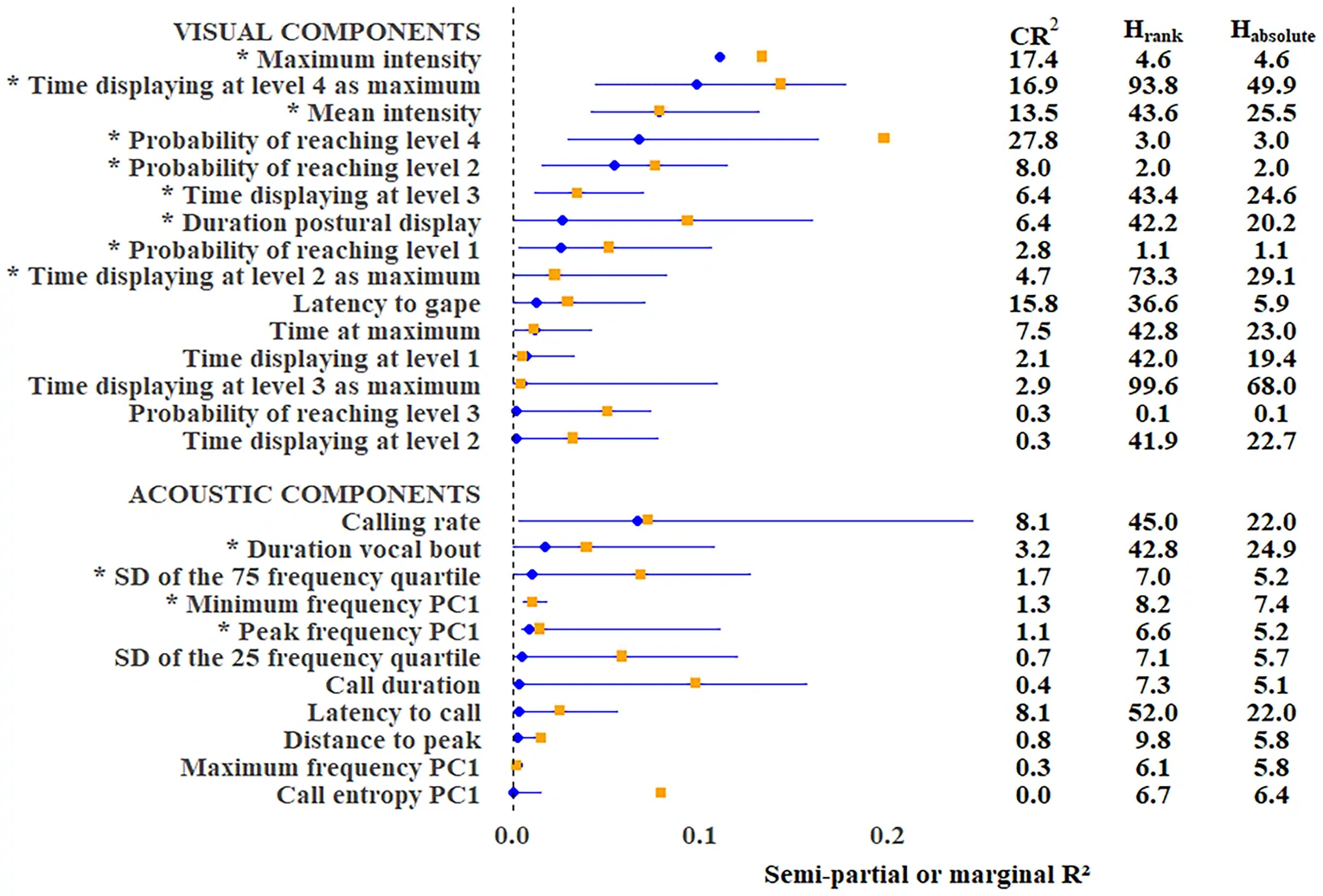
Effectiveness of postural and acoustic begging components in encoding deprivation time in laboratory nestlings. Blue bars show semi-partial *R*² (± 95% CI), representing variance uniquely explained by deprivation time; yellow points indicate marginal *R*² (fixed effects). Effectiveness is also expressed as the percentage of total explained variance (fixed + random effects) attributable to deprivation time (semi-partial/conditional *R*² ratio, CR2) and as percentage uncertainty reduction (H) under two discretization methods. * corrected *p*-value < 0.05

Naturally, MI was lowest for components with discrete values (e.g. maximum postural intensity and the probability of reaching a given intensity level) and higher for continuous variables discretized as percentiles. For continuous traits, MI was consistently greater when discretization was based on rank (equal-frequency bins) rather than on absolute value ranges, especially for temporal variables. The time spent at intensity levels 2, 3 and 4 (as maxima) produced the largest reductions in uncertainty. Postural display duration conveyed a comparable amount of information, but its semi-partial coefficient of determination had confidence intervals overlapping zero. In this case, most of the variance was attributable to random effects (nestling and natal brood identity), whereas deprivation time explained only approximately 2% of the variance (figure 4). Overall, temporal components of the postural display followed a common pattern: relatively high uncertainty reduction (41.9–99.6% under rank-based discretization) but a low fraction of explained variance (0.2–6.4%), with notable exceptions for time spent at level 4 and latency to beg, which showed high semi-partial-to-conditional *R*^2^ ratios comparable to those of maximum postural intensity (figure 4).

Vocal components were generally less effective than postural components. Confidence intervals for semi-partial R^2^ overlapped zero for all acoustic variables except minimum and peak frequency PC1 and the s.d. of the frequency P75. Only calling rate, latency to call and vocal-bout duration achieved uncertainty-reduction values comparable to those of postural components (figure 4). Among these, latency to call was the only trait for which deprivation time accounted for a relatively large fraction of the variance relative to other fixed and random effects.

Figure 5 summarizes receiver-based decoding analyses showing how predictive accuracy for deprivation state changes as a putative receiver integrates progressively more components during a begging bout. In all three panels (alternative predictor orderings), accuracy increased sharply as soon as the first front-line cues were available—latency and maximum postural intensity—reaching values well above the baseline expectation (dotted line). Thereafter, adding further mid-bout components produced only modest gains and, in some cases, a transient drop toward baseline, indicating substantial redundancy once these early cues were included. The main divergence among curves occurred at steps 3 and 4, depending on whether the calling rate was entered before the duration at maximum intensity (orderings based on semi-partial *R*^2^, figure 5*a* and rank-based MI, figure 5*b*) or vice versa (ordering based on MI from absolute-value discretization, figure 5*c*): only in the latter case did the calling rate yield an immediate increase in accuracy. Despite this order dependence at intermediate steps, the curves converged once both components had been included, consistent with substantial overlap (redundancy) in the information conveyed by most acoustic predictors. Finally, predictive accuracy increased again when late-available bout-level metrics were added (figure 5). Notably, total bout duration produced a substantial improvement despite entering last. Because late-entering variables are inherently penalized in cumulative models—their incremental effect is assessed only after correlated predictors have already been included—this pattern indicates that receivers can further refine their estimate of deprivation once the display has fully unfolded.

**Figure 5.**
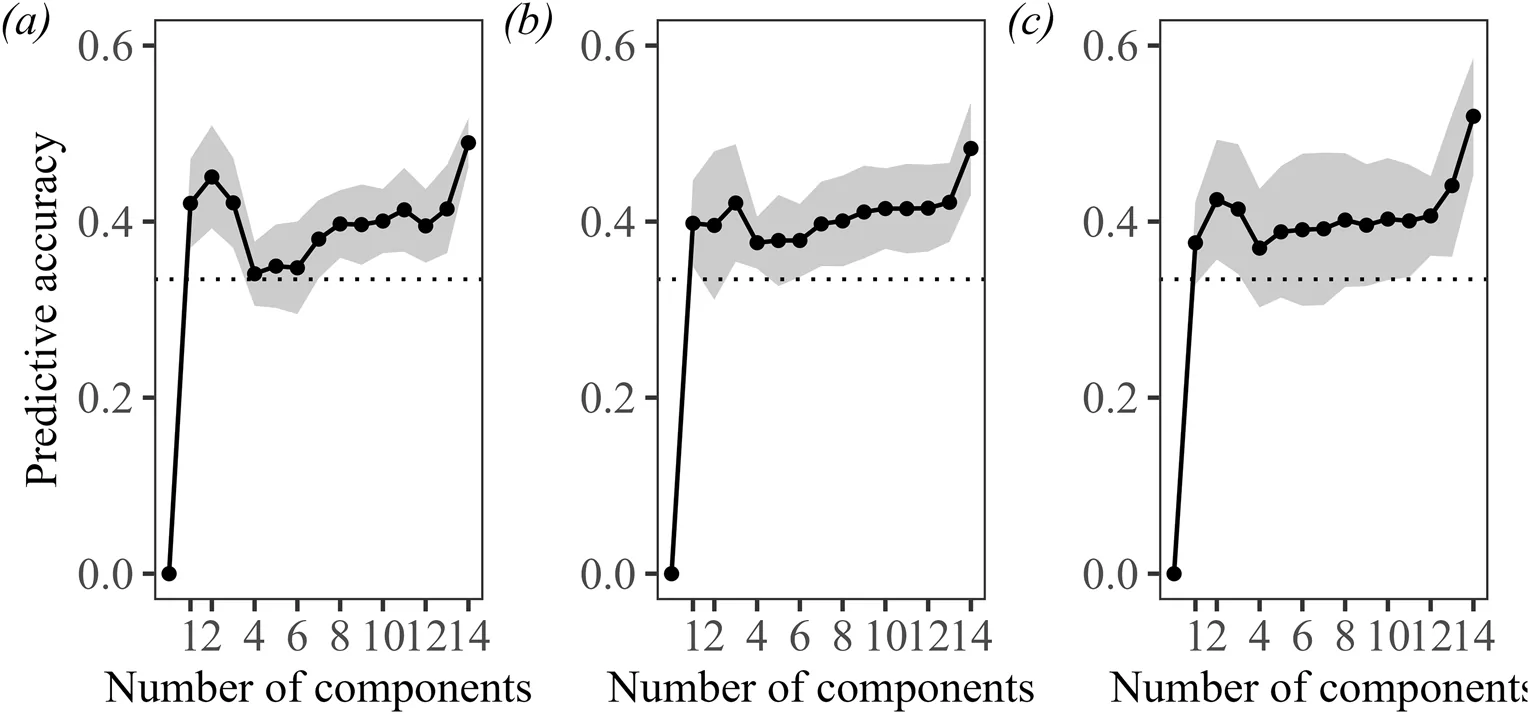
Receiver-based decoding of deprivation state from laboratory begging bouts. Lines show mean (± s.d.) grouped K-fold cross-validated random-forest accuracy as components (1–14) are added sequentially during a bout. (*a*) Components are ordered by decreasing semi-partial *R*², (*b*) rank-based uncertainty reduction and (*c*) absolute-scale uncertainty reduction. Acoustic latency enters first, maximum postural intensity second, and calling-bout duration last.

#### 3.2.4. Effectiveness of postural begging as a discrete coding scheme

Figure 6 summarizes signal-effectiveness measures for postural intensity treated as a discrete coding scheme, showing how performance changes with the number of signal categories (pools) in both wild broods and laboratory nestlings. For wild broods, it also contrasts two ways of discretizing continuous deprivation time: a rank-based approach and an absolute-scale approach.

**Figure 6.**
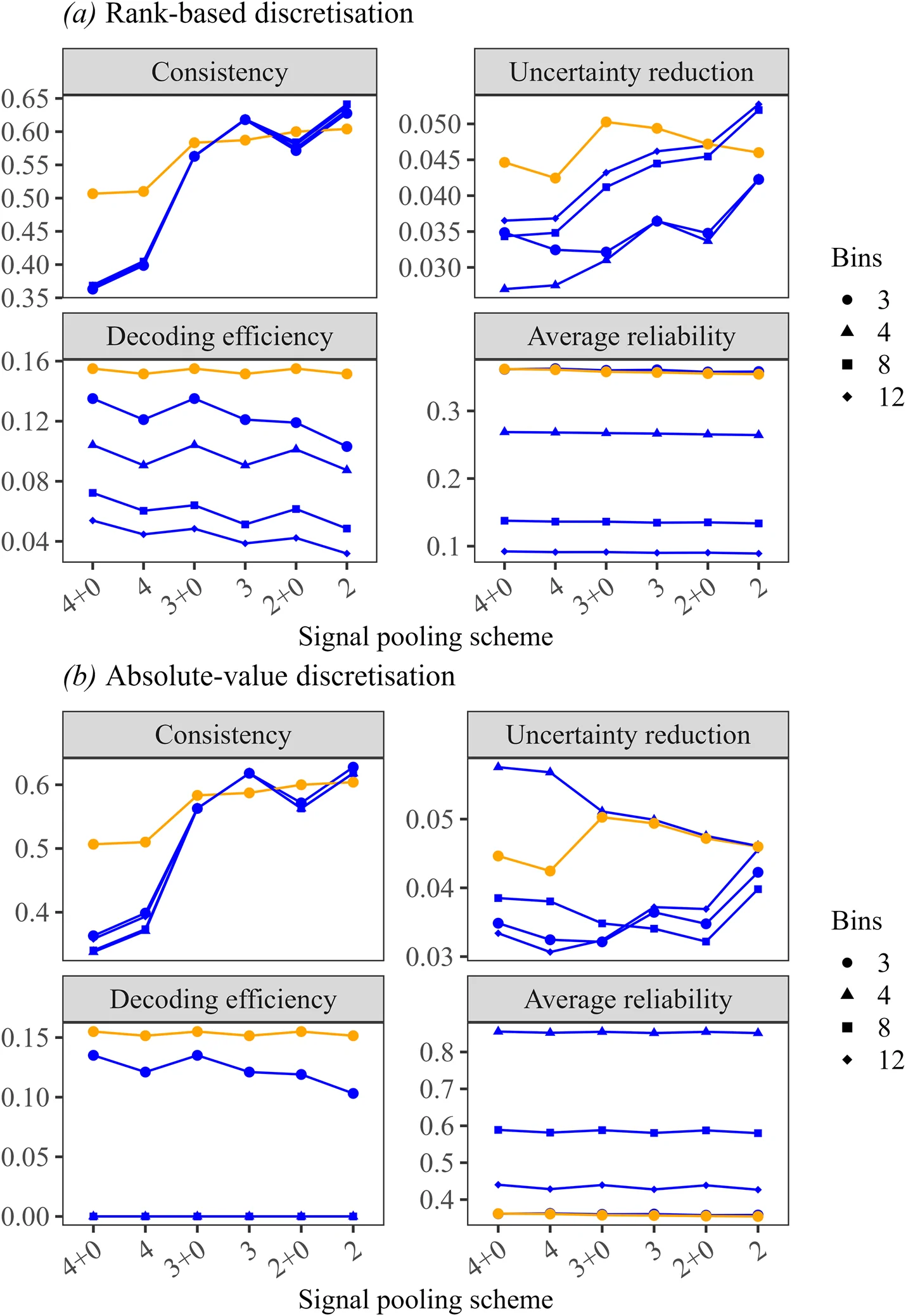
Effectiveness of postural begging intensity as a discrete coding scheme. Postural levels were grouped into alternative pooling schemes (four to two pools), with or without a non-begging category (‘+0’). Panels show (left to right and top to bottom) average consistency (regularity of signal production under a given deprivation state), uncertainty reduction (fraction of initial uncertainty removed), Bayes decoding efficiency and average reliability (two receiver-performance measures). Laboratory nestlings are shown in yellow and natural broods in blue. Field data are evaluated under different deprivation-time resolutions (3, 4, 8 and 12 bins) and discretization methods ((*a*) rank-based; (*b*) absolute-scale).

From a signaller's perspective, the simplest coding scheme was also the most consistent in both settings, regardless of discretization method: a two-category code contrasting ‘down’ versus ‘up’ postures (figure 6*a*,*b*; upper left panels). In laboratory nestlings, however, uncertainty reduction was maximized by a coding scheme that distinguished three postural categories (gaping while lying with the neck flexed; gaping while lying with the neck extended; gaping upright) and included a non-signalling state; performance was lowest under the most complex schemes that retained four postural levels, with or without a non-signalling category (figure 6*a*,*b*; upper right panels). In wild broods, where deprivation varied continuously, uncertainty reduction was highest—given any fixed number of deprivation bins—under the simplest two-category posture code when deprivation was discretized by rank (levels 1+2 pooled versus 3+4 pooled). A similar pattern emerged under absolute-value discretization, except when deprivation was divided into four categories, in which case the most informative code was the most complex one (figure 6*b*; upper right panel).

From a receiver's perspective, Bayes-normalized decoding efficiency was highest when posture encoded coarse deprivation states (the three-bin scheme) and was relatively insensitive to the number of postural signal categories, although it peaked slightly for the scheme that pooled posture into three categories plus a non-signalling state (figure 6*a*,*b*; lower left panels). Under absolute-value discretization, subdividing deprivation time into finer categories (four bins or more) reduced decoding efficiency to near zero; in practice, observing the signal did not improve classification beyond always choosing the most frequent deprivation category. This reflects the strongly right-skewed distribution of deprivation times in natural broods, with most observations concentrated at short intervals. Consequently, a naive decoder that assigns the dominant short-deprivation state achieves a high baseline accuracy that approaches the Bayes optimum, leaving little scope for the signal to improve performance unless it mainly encodes coarse absolute differences (or relative ranks) in deprivation.

Average reliability (figure 6*a*,*b*; lower right panels) varied little across pooling schemes in both wild broods and laboratory nestlings: more complex codes were not consistently more reliable than simpler ones. This becomes clear when examining the distribution of reliability across specific signal–state combinations. Assuming that posture encoded three biologically meaningful deprivation states, displays were most reliable at indicating the extremes (very low or very high deprivation), whereas they provided comparatively little reliable information about intermediate deprivation levels (table 7). Interestingly, reliability distributions were strikingly similar between wild and laboratory settings despite a four-fold difference in absolute deprivation times. In both cases, parents could reliably infer that a chick that did not beg or begged at level 1 had not been deprived for long, but could not discriminate among very short intervals. Short deprivation was most consistently indicated by level 2 begging, whereas long deprivation was most reliably signalled by level 4 displays (table 7). In wild broods, average reliability was highest under coarse discretizations of deprivation (three or four bins, depending on the discretization method).

**Table 7.** Receiver inference accuracy (reliability) for postural begging levels as discrete signals in wild and laboratory nestlings. Shown are the ratios of posterior (updated) to prior probabilities for each deprivation state after observing a given postural level.

	postural intensity				
**deprivation time (min)**	**not gaping**	**1**	**2**	**3**	**4**
**wild**					
low (< 8.5)	0.49	0.52	1.06	0.73	0.74
medium (8.5 < t < 25.5)	0.22	0.24	0.85	0.57	0.91
high (>25)	0.17	0.11	0.52	0.72	1.26
**laboratory**					
30	0.03	0.15	1.77	0.51	0.54
60	0.03	0.09	1.38	0.45	1.05
90	0.00	0.00	1.02	0.57	1.41

## 4. Discussion

### 4.1. Summary of main results

In wild broods, both begging order and maximum postural intensity increased with longer deprivation times, but deprivation time explained far more variance than prey size. Yet the information encoded was modest: between 3% (MI) and 8% (semi-partial *R*^2^) for maximum postural intensity, the most effective component. Parents typically remained in the nest for 12–17 s, but most feedings were delivered within the first 5 s after arrival. They favoured chicks that begged first, while postural intensity increased the probability of being fed among nestlings begging later in the visit. In addition, parents relied on non-signal cues, including nestling mass and nest position, when allocating food.

Analyses of laboratory begging components identified maximum postural intensity as the cue explaining the largest share of variance. Time spent at, and the probability of reaching, postural level 4, mean intensity and latency to gape followed closely, showing the highest semi-partial effects. By contrast, total display duration conveyed similar MI but little unique variance, as most of its variation was attributable to nestling and brood identity rather than deprivation. Acoustic variables were generally weaker. Only calling rate, latency to call and vocal-bout duration reached uncertainty-reduction values comparable to the weaker visual traits, and only latency to call explained a substantial fraction of variance relative to other fixed and random effects. Spectral features contributed negligibly. MI revealed the same hierarchy, although it was necessarily lower for inherently discrete traits and higher for continuous variables. Uncertainty reduction was consistently higher under rank-based than under absolute-scale discretization, reflecting the highly skewed distribution of natural deprivation times and suggesting that relative, rather than absolute fine-scale, variation in deprivation is more relevant for the interpretation of begging displays.

Receiver-based decoding analyses showed that most information about deprivation was available early in the begging bout. Predictive accuracy increased sharply once latency and maximum postural intensity were included, surpassing baseline expectations after only one or two cues. Adding further mid-bout components produced little additional improvement, indicating substantial redundancy once these initial cues were known. It remains possible, however, that unmeasured traits (e.g. amplitude) are informative, or that parents integrate acoustic cues in a non-additive, multi-variate manner [89]. Predictive accuracy rose again when late bout-level measures were incorporated, particularly total bout duration, which produced a marked gain despite entering last.

Postural levels in both wild and laboratory nestlings overlapped extensively in associated deprivation states, conveying only coarse information rather than precise estimates of deprivation time. Laboratory analyses of display structure likewise showed little evidence for a graded, information-rich signal: nestlings reached maximum intensity at the start of the display and then relaxed into lower levels in a stereotyped sequence. Only levels 2 and 4 differed consistently between weak and strong deprivation, and time spent at submaximal levels did not encode intermediate hunger; only duration at level 4 increased reliably with deprivation. Discrete coding analyses of the postural display further supported a pooling rather than a graded coding scheme. Although, as in all previous studies, our interpretation is constrained by the use of ordinal rather than continuous objective measures of body stretching, signal effectiveness did not increase monotonically with the number of postural pools. This pattern was consistent across all measures of coding performance. In both wild and laboratory nestlings, the simplest two-category scheme (‘down’ versus ‘up’) was the most consistent from the signaller's perspective. In laboratory nestlings, uncertainty reduction peaked with three postural categories plus a non-signalling state, whereas the most complex four-level schemes performed worst. Similarly, in wild broods—where deprivation varied continuously—uncertainty reduction was generally highest under two-category pooling, particularly when deprivation was rank-discretized. From the receiver's perspective, Bayes decoding performance and average reliability varied little across pooling schemes and were highest when posture encoded coarse deprivation states. Finer absolute-scale discretization of deprivation time in wild broods often reduced decoding performance to baseline because most observations clustered at short intervals. In both field and laboratory data, displays reliably indicated extreme hunger (very low or very high deprivation) but conveyed little information about intermediate states, and reliability patterns were strikingly similar despite a four-fold difference in absolute deprivation times.

### 4.2. Comparison with previous studies

Previous studies on begging honesty have not quantified signal effectiveness in the way implemented here, limiting direct comparison. Nonetheless, our results align well with patterns reported across many avian studies (tables 8 and 9). The literature shows strikingly consistent trends: postural intensity increases with deprivation and declines after feeding (32/33 studies); deprivation lengthens postural displays (12/13), increases total vocal output (sometimes termed ‘begging rate’, 24/27), and elevates call amplitude (16/22). Many studies accounted for random intercepts (tables 8 and 9), underscoring the robustness of these relationships. Few studies have examined how deprivation affects specific postural levels or the fine temporal structure of visual displays. One exception is Savagian & Riehl [90], who found in greater anis (*Crotophaga major*) that maximum postural intensity reflected hunger chiefly through a contrast between highest-intensity begging and no begging—consistent with our finding that posture codes effectively only for extreme states.

**Table 8.** Reported effects of hunger (deprivation time or food intake) on postural begging variables across studies. Entries indicate whether each study found a statistically significant positive (+), negative (–) or non-significant (0) effect and the statistical method used.

species	intensity	duration	latency	gaping order	statistics^1^	reference
**deprivation time**						
*Agelaius phoeniceus*	+		–		LMM	[91]
*Cercotrichas galactotes*	+			–	other	[92]
*Crotophaga major*	+				LMM	[90]
*Cyanistes caeruleus*		+			LMM	[93–95]
*C. caeruleus*	+	+	–		LMM	[96]
*C. caeruleus*	+	+			LMM	[63]
*Ficedula hypoleuca*	+	+			LMM	[97]
*F. hypoleuca*	+			0	other	[98]
*F. hypoleuca*	+	+	0		LMM	[99]
*F. hypoleuca*	+			–	LMM	[65]
*Hirundo rustica*	+				LMM	[32,100]
*Megascops asio*	+			–	other	[101]
*Molothrus badius*	+	+	–		LM	[102]
*Parus major*	+				LMM	[103]
*Pica pica*	+			–	LMM	[104]
*P. pica*	+				other	[105]
*Platycercus elegans*	+				LMM	[106]
*Ploceus jacksoni*	+				LMM	[50]
*Serinus canaria*	+				LMM	[107,108]
*Setophaga petechia*	+			–	LM	[109]
*Sialia mexicana*	+				LMM	[51]
*Tachycineta bicolor*	+			–	LM	[110]
*T. bicolor*	+				other	[111]
*T. bicolor*	+	+	0		LMM	[112]
*Turdus migratorius*	+			–	other	[75]
*Turdus rufiventris*	+			–	LM	[109]
*Upupa epops*	+	+			LMM	[113]
*Xanthocephalus xanthocephalus*				–	LM	[114]
**Food amount**						
*C. galactotes*	0			0	other	[92]
*C. major*	–				LMM	[90]
*Falco tinnunculus*		–			LMM	[115]
*Phoenicurus phoenicurus*				+	other	[116,117]
*P. pica*	–	0		+	other	[44]
*Sturnus vulgaris*	–			+	LM	[118]

^1^ LM: linear model, LMM: linear mixed model; other: uni- or bivariate analysis not including interaction effects.

**Table 9.** Reported effects of hunger (deprivation time or food intake) on acoustic begging variables across studies. Entries indicate whether each study found a statistically significant positive (+), negative (–) or non-significant (0) effect and the statistical method used.

species	vocal output^1^	calling rate^2^	latency	call duration	amplitude	frequency	Frequency range	call entropy	statistics^3^	reference
**deprivation time**										
*Aethia cristatella*	+	+				+		+	LMM	[119]
*Aethia psittacula*	+	+				+		+	LMM	[119]
*Anthus pratensis*	+			0	0	0			other	[120]
*A. pratensis*	+				+		+		LMM	[121]
*Calonectris leucomelas*	+	+							LMM	[122]
*Cyanopica cooki*	+		–	+	0	+			other	[123]
*Fratercula corniculata*	+	+				+		+	LMM	[119]
*Gerygone igala*	0	0			–	0	–		LM	[77]
*Grus leucogeranus*	+					+		+	LMM	[124]
*H. rustica*					+				LMM	[32,125,126]
*H. rustica*	0			0	+				LM	[127]
*H. rustica*	+				+				LM	[128]
*H. rustica*	0	0	0	+		0			LM	[129]
*Larus delawarensis*	+				+				LM	[45]
*Larus glaucescens*	+				+				other	[130]
*M. asio*		+			+				other	[101]
*P. major*					+				LM	[131]
*Passer hispaniolensis*	+		0	0	+	+			other	[132]
*P. pica*	+				0				other	[105]
*P. jacksoni*	+			+	+	+		+	LMM	[50]
*Prunella modularis*	+			0	0	0			other	[120]
*T. bicolor*	+			+	+	0	0		other	[133]
*T. bicolor*	+			+	+	0	+		LM	[134]
*T. bicolor*		0							other	[111]
*T. bicolor*	+	+	0						LMM	[112]
*X. xanthocephalus*	+				+				LM	[135]
**food amount**										
*C. leucomelas*	–	0							LMM	[122]
*L. delawarensis*	–				0				LM	[45]
*Puffinus puffinus*	–	–							other	[136]
*Troglodytes aedon*	–								LMM	[137]

^1^ Total calling time or calling rate measured over a period longer than the average calling bout.

^2^ Within-bout calling rate, measured over a period equal to or shorter than the average calling bout or quantified as the interval between successive calls within a bout.

^3^ LM: linear model; LMM: linear mixed model; other: uni- or bivariate analyses without interaction terms.

Findings for other temporal components are more mixed, again matching our results. In spotless starlings, latency to gape was significantly related to deprivation only when chicks were immune-challenged [138], suggesting that reactiveness may also reflect nestling health or vigour [139]. No previous study has tested whether latency varies with recent food intake, and only four of nine studies reported an effect of deprivation on postural or vocal latency (tables 8 and 9). In some cases, the relationship was nonlinear or bimodal, with longer latencies occurring only at very short deprivation times [91,102,140]. Even such coarse variation may help explain the strong association between begging order and both deprivation time (9/10 studies) and food intake (4/5 studies) (table 8).

Similarly, results for calling rate and call duration vary across studies: 7 of 11 found a positive effect of deprivation on within-bout calling rate, and five of nine reported increased call duration with hunger. Evidence for spectral variables is also inconsistent: 7 of 13 reported a positive association between call frequency and deprivation, and findings for frequency range were equally heterogeneous (table 9).

Seemingly supportive results, particularly for acoustic parameters, must be interpreted cautiously for two reasons. First, call rate and frequency depend on nestling mass [141], sex [135,142] and temperature [133], yet few studies have examined these factors jointly with deprivation. Exceptions include [50,121,127] for mass and sex; [123,133 for mass and temperature; and [129] for brood size and mass. Because these factors interact with hunger, not controlling for them can overestimate the main effects of deprivation [47,76,135]. Second, although some studies used random intercepts, none included random slopes, which can inflate both type I and type II errors [143]. In our analyses, omitting random slopes substantially inflated inference, increasing the number of acoustic variables spuriously inferred to encode deprivation from three to six or seven, depending on the *p-*value adjustment applied.

### 4.3. Hunger signals need not be precise to be functional

Nestling begging influences both food allocation and overall provisioning by parents [144]: intensely begging nestlings receive more food [145], and elevated begging increases parental effort at the brood level [146]. In species raising multiple chicks, parents typically deliver only one or a few items per visit (94% in our sample), so they need only identify the relatively most deprived chick(s), not precisely assess each nestling's condition. When receivers make discrete decisions (feed or not), theory predicts that they classify signallers into broad categories [147,148], as in pooling equilibria [20], and perceptual error, which may be substantial in multi-chick broods, further favours binary, all-or-nothing signals [149]. Our results indicate that parents can make accurate relative assessments simply by observing which chick begs first and whether it fully stretches or not. For postural intensity, binary schemes were, on average, the most consistent, which is noteworthy because any benefit of adding more postural categories must be weighed against the time or cognitive costs of discriminating among highly overlapping levels [150]. Such costs would diminish the value of more complex but inconsistent coding schemes. More finely tuned cues—such as subtle changes in mouth colour—may evolve when food can be divided [151], although even in those cases parents often need only decide which chick receives the first, and usually largest, portion [75,152]. Allocation based on relative rather than absolute, fine-scale variations in hunger may be particularly efficient for spotting the most deprived chick under fluctuating feeding schedules [153], as commonly observed within days [154,155] and between days [156,157]. Consistent with this, postural begging was coded similarly in the laboratory and field despite a four-fold difference in mean deprivation times. Such flexibility may be facilitated by learning [29,158,159], a form of behavioural plasticity that is not easily accommodated within traditional signalling models [49].

In multi-chick broods, nestlings compete during parental visits to obtain one or a few feedings. Because parental allocation decisions occur rapidly after arrival at the nest, a signal that quickly directs parental attention to a particular chick may be highly effective even if it conveys only coarse information about hunger. Parents also use non-begging cues when allocating food, including nestling mass and nest position [65]. This should limit parental attention to any single begging component, creating a situation akin to strategic receiver inattention [160]. Under such conditions, highly conspicuous early components of begging may function partly as attention-seeking displays [42], helping parents locate or select a chick rapidly before any finer assessment of need occurs. Several of our findings are consistent with this possibility. Receiver-decoding analyses showed that most predictive information became available immediately through latency and maximum postural intensity, with subsequent components adding relatively little information. Likewise, nestlings typically reached maximum postural intensity almost immediately and then relaxed into lower postures, a temporal pattern that resembles alerting or attention capture more than a finely graded coding system. The extensive overlap among deprivation states and postural categories may, therefore, reflect not only pooling but also optimization for conspicuousness rather than precise coding of deprivation time. In that case, low information content would not be a deficiency of the signal, but a consequence of some components serving a different function.

Parental provisioning can also remain sensitive to brood-level hunger even when individual signals are only partly informative. In multi-chick broods, parents may assess gradations in collective need by integrating the additive effects of simple, all-or-nothing displays—for example, by responding to the number of chicks that gape or call [7]. Provisioning effort scales with brood size [161] and often increases in response to collective begging—particularly when more gapes are visible or when brood-level vocalizations intensify—both of which independently elevate parental effort [162]. Although postural intensity has no effect in single-chick experimental set-ups [7], some studies report effects in multi-chick broods (e.g. [163–165]), but these may simply reflect variation in the number of nestlings gaping simultaneously. Begging calls alone can function as group-level signals [166], influencing provisioning in several species [167–169], yet little is known about parental responses to fine-scale acoustic structure beyond call rate [170,171] or about their ability to discriminate individual calls within a noisy brood. After delivering food, spotless starling parents typically remained in the nest for longer than the duration of the entire begging display [19]. Begging during this non-feeding phase may yield delayed benefits to nestlings [60,172] if parents use additional information—such as collective display duration or call amplitude—to adjust subsequent provisioning effort [114,146]. This mechanism may explain why nestlings continue begging beyond the time required to secure the immediate feeding.

Summarizing, begging displays appear to provide partly honest information about coarse differences in deprivation—and secondarily about recent food intake—rather than fine-scale individual variation. Our results challenge the prevailing view that postural begging is a graded, information-rich signal of the type predicted by separating-equilibria models [11,12,18]. Instead, postural begging appears more consistent with a discrete system comprising few, highly overlapping categories, as predicted by pooling-equilibria [14,20,37], or with an attention-seeking display whose primary function may be to secure parental attention rather than to encode deprivation precisely [42,159]. Continuous acoustic traits likewise contributed little to the information content of the multi-component display, calling into question the assumption that begging is information-rich—and therefore beneficial to receivers—simply because it spans multiple sensory modalities [173]. Alternatively, acoustic components may also enhance detectability in multi-chick broods rather than convey detailed information to parents [111,134,174] or mediate communication between nestlings [32,168].

Several avenues for future research follow from these results. First, the generality of our conclusions should be tested across a broader range of bird species. The metrics used here are well-suited to interspecific comparisons and could reveal how signal effectiveness varies with ecological and life-history traits, including brood size, nest type and provisioning regime. For example, parents of open-nesting species may rely more on visual displays during food allocation than those of cavity-nesting species [111,171]. Attention-seeking may also be less important in single-chick broods, whereas slow provisioning rates may limit parents' ability to track rapid changes in offspring hunger [171]. Future studies should also quantify not only the effectiveness of each begging component, but also the extent of apparently random variation and whether it follows patterns consistent with mixed or hybrid equilibria [41]. Second, the function of the substantial variation in acoustic begging remains poorly understood, and several possibilities require formal testing. Acoustic output may convey collective brood demand for provisioning [172,175], mediate cooperative–competitive interactions among nest-mates in multi-chick broods [176,177] or reflect aspects of nestling condition other than hunger [178].

## 5. Conclusion

Nestling begging in spotless starlings is not a finely graded, information-rich signal of individual hunger, as traditionally assumed, but a partly informative system better described by pooling-equilibria or attention-seeking models. This conclusion emerges from both random-regression and information-theoretic analyses, approaches that have not previously been applied in such depth to begging displays, and it is broadly consistent with earlier empirical findings. Parents nonetheless make effective allocation and provisioning decisions by integrating a small set of robust, low-resolution signals across individuals and time. These results reconcile the apparent effectiveness of begging with its low information content and limited costs, and highlight the need to move beyond assumptions of precise, costly honest signalling toward frameworks that emphasize imprecise coding in parent–offspring communication.

## Supplementary Material



## Data Availability

The data and code that support the findings of this study are openly available at DIGITAL.CSIC repository [179]. Supplementary material is available online [180].
