# Supplementary material for: Effectiveness of multi-component, dynamic begging displays as honest signals of nestling hunger
Source: R Soc Open Sci. 2026 Oct 14;13(10):260413. doi: 10.1098/rsos.260413 (PMC13682431; doi:10.1098/rsos.260413)

Supplementary Material for *Effectiveness of multicomponent, dynamic begging displays as honest signals of nestling hunger*

Tomás Redondo[1]* and Daniel Parejo-Pulido[2]

[1] *Estación Biológica de Doñana (EBD), CSIC, Américo Vespucio 26, 41092 Sevilla, Spain.*
[2] *Instituto de Investigación en Recursos Cinegéticos (IREC), CSIC-UCLM-JCCM, Ronda de Toledo 12, 13005 Ciudad Real, Spain.*

Supplementary Methods

1. Nestling husbandry and feeding protocols at the laboratory

On the afternoon prior to the experiment, we selected two five-day-old chicks of intermediate mass rank from each brood (27 males and 23 females; mass range: 22.9–32.8 g). To avoid parental desertion, at least two chicks remained in each brood; in broods of two or three chicks, temporary replacements from other broods were used. Selected nestlings were transported in a warm container to the laboratory and placed together in artificial nest cups under controlled temperature (≈35°C) and humidity (≈50%).

The following morning at 08:00, nestlings were weighed (excluding faecal sacs) using a digital balance (0.1 g accuracy), then placed individually into separate artificial nests and fed every 45 min until satiation. Voluntary intake at satiation is a reliable proxy for begging intensity [1]. Feeding intervals followed estimates of natural parental feeding rates from continuous recordings in 48 nests (mean interval = 59.1 ± 4.27 min SE; inter-feeding interval = 60/N, where N is the number of feedings per chick per hour).

Food quality is also known to influence begging behaviour [2], and voluntary intake may also depend on long-term nutritional condition [3] or variation in nutrient reserves among nestlings [4]. We therefore provided a varied diet during the four-trial conditioning phase to minimise potential baseline differences in nutritional state. Chicks received tiny pieces of boiled egg (trials 1 and 4), *Zophobas morio* superworms (trial 2), and *Acheta domestica* crickets (trial 3). Ration sizes were calculated using Weathers' [5] allometric equation for daily food intake:

$$\text{Daily intake} = 0.98\ M^{0.814},$$

where M is body mass (g), assuming 14 daily portions (mean insect ration: 3.23% ± 0.15 SE of body mass; boiled egg: 3.5%).

Before the begging trials, all nestlings received 3.5% of their body mass as high-quality food (boiled egg; [6]) to standardise intake relative to body mass. Consequently, variation in hunger during the experiment primarily reflected differences in the duration of food deprivation rather than short-term differences in food intake.

2. Acoustic analysis of begging calls

We analysed begging vocalisations using Avisoft SAS-Lab Pro v.5.1.23 [7]. Begging calls consisted of multiband thrills with varying degrees of frequency modulation (Supplementary Figure S1). Spectrograms were generated with the following settings: 256-point FFT, FlatTop window, 100% frame, 50% overlap, 162-Hz bandwidth, and a 2-kHz high-pass filter, yielding a time resolution of 11.6 ms and a frequency resolution of 43 Hz.

From these spectrograms, we extracted temporal and spectral measurements. Total vocal bout duration (ms) was measured manually from the onset of the first call to the end of the last, and the number of calls and latency to vocalise (time from stimulus onset to the first call) were recorded. Calling rate was computed as the number of calls divided by bout duration.

For spectral analyses, we selected up to the first 10 calls in each bout. Individual calls were automatically detected using amplitude thresholds and manually verified to ensure correct segmentation. For each call, we obtained automatic measurements of duration, time to the point of maximum amplitude, peak frequency, higher and lower frequencies, and entropy. Entropy quantifies the distribution of energy across the spectrum, with pure tones exhibiting low values and broadband signals higher ones.

Because spectral measurements in multiband, frequency-modulated calls can vary markedly across adjacent time segments, entropy and frequency variables were measured at four points: (i) two at the point of maximum amplitude—one instantaneous and one using a 40-ms minimum hold (peak-hold mode), and (ii) two derived from the Max and Mean spectra of the entire element. For elements combining constant-frequency and frequency-modulated sections, the Mean spectrum is dominated by the constant component, whereas the Max spectrum shows a flat-top profile spanning the full frequency range of the syllable [7]. For whole-element measures, robust estimates of low and high frequencies [8] were obtained using the 25th (P25) and 75th (P75) frequency percentiles rather than instantaneous extrema. Frequency modulation was quantified as the standard deviation of these two frequency percentiles within each call.

In total, 1,904 calls were analysed. To integrate the four sets of spectral measures obtained at different time points, we performed separate Principal Components Analyses for peak frequency, maximum frequency, minimum frequency, and entropy. In all cases, PC1 accounted for 64–73% of variance (Supplementary Table S1), and PC1 scores were used in statistical analyses. We checked that results from univariate analyses of individual spectral variables (unreported) were consistent with those based on PC1 scores. Acoustic begging could not be recorded for the first two nests.

3. Statistical analysis

3.1. Ordinal variables in natural broods

To standardise begging-order ranks across broods of different sizes, we transformed raw ranks following Smith and Montgomery [9] using the formula (value−1) / (brood size−1), such that 0 corresponds to the first nestling to beg. Spatial profitability was calculated as in Parejo-Pulido et al. [10] and similarly standardised across brood sizes following Smith and Montgomery [9], with 0 indicating the most profitable nest position.

3.2. Structure of mixed models

For each response variable, model selection followed a two-step procedure in which the random-effects structure was optimised prior to fixed-effects selection [11]. Analyses were conducted in R using mixed-effects models fitted with the lme4 package, with Gaussian responses analysed using linear mixed models (*lmer*) and binary responses analysed using generalized linear mixed models with a binomial error distribution and logit link (*glmer*) [12]. When the response variable was ordinal, we additionally fitted cumulative link mixed models with a logit link using the ordinal package [13], retaining the optimal random-effects structure identified when the response was treated as Gaussian.

For random-effects selection, we began with a maximal structure reflecting the full hierarchical design of the data (nest, chick or parent, and feeding sequence) [14] and simplified it by sequentially removing random components while keeping the fixed-effects structure constant. For Gaussian mixed models, alternative random-intercept and random-slope specifications were compared using information-theoretic criteria (AIC), implemented via the model.sel() function in the MuMIn package [15]. For binomial generalized linear mixed models, competing random-effects structures were compared using likelihood-ratio tests based on nested model comparisons (anova), fitted without fixed effects [11].

Once the optimal random-effects structure had been identified, fixed-effects selection was conducted using an information-theoretic approach. Starting from a biologically motivated global model including all main effects and interactions of interest, we generated all possible submodels and ranked them using the dredge() function in MuMIn, comparing models based on AICc. In all cases, the models with the lowest AICc retained the main effect of deprivation.

Model assumptions were assessed visually by checking residuals using the performance package [16] for linear mixed models and DHARMa [17] for generalized mixed models.

3.3. *Controlling false discovery rates*

To account for multiple statistical tests across different aspects of begging behaviour, we controlled the false discovery rate (FDR) using the Benjamini–Hochberg (BH) procedure [18,19], which offers a more balanced control of type I and type II errors than the Bonferroni correction [20,21]. Response variables were grouped according to the hypotheses addressed: (1) overall visual begging performance in the laboratory (latency, duration, mean and maximum postural intensity); (2) decomposed postural intensity components in the laboratory; (3) acoustic begging variables; and (4) postural begging intensity in natural broods. The BH correction was applied independently within each group. When an interaction term became non-significant after correction, the procedure was repeated including the P-value of the corresponding main effect.

3.4. Measures of overlap and effectiveness in discrete coding schemes

To quantify the extent to which different begging-intensity levels overlapped in the deprivation states they were associated with, we compared the empirical distributions of deprivation time for every pair of postural levels. For each postural level in natural broods, we estimated a smoothed kernel density of deprivation time using the base R function density(), with its default data-driven bandwidth (Supplementary Figure S2). For each pair of postural levels, the two density curves were evaluated on a common grid, and their overlap coefficient (OVL) was computed as the integral of the pointwise minimum of the two densities, numerically approximated as:

$$OVL = \sum[min(d1(y), d2(y))]\ \Delta x,$$

where d_1 and d_2 are the outputs of density() and Δx is the grid spacing. This was implemented using base R functions and dplyr package for data handling [22]. OVL ranges from 0 (no overlap) to 1 (complete overlap), providing a scale-free measure of how distinct the deprivation-time distributions are across postural intensity levels.

To compute signal effectiveness, we constructed sender coding matrices using contingency tables of frequencies of different maximum postural levels observed under each pooling scheme and deprivation condition, with postural levels and deprivation conditions, respectively, varying across pooling schemes and discretisation bins. In natural broods, we discretised continuous deprivation times using multiple bin sizes (4, 8, 12 and 16 bins) and additionally defined three biologically motivated deprivation categories comparable to those used in the laboratory experiment. Our rationale was as follows. Consider a parent provisioning a brood of N chicks at a constant rate, delivering one prey item every t minutes. The mean deprivation time (t_m) for the brood is:

$$t_m = \frac{t}{N}\sum_{i=1}^{N} i$$

At any given visit, parents do not know precisely how long each chick has been deprived but should benefit from identifying those with the most extreme deprivation times. In a brood of four chicks, the most deprived chick is four times more deprived than the least deprived chick and 1.6 times more deprived than the average chick (2.5 t). In a brood of five chicks, the most deprived chick is 1.7 times more deprived than the average chick (3 t). Conversely, the two least hungry chicks experience feeding intervals of roughly 0.6 t_m (four chicks) or 0.5 t_m (five chicks).

From this, we assumed that parents are most interested in nestlings whose deprivation times are either at or above $1.5 \times t_m$ or at or below $0.5 \times t_m$. Using the mean deprivation time in our sample (17 min) as a reference, we therefore discretised natural-brood deprivation times into three categories comparable to the 30, 60, and 90 min conditions in the laboratory experiment: short (time ≤ 0.5 × 17 min; N = 906 observations), long (time ≥ 1.5 × 17 min; N = 1156), and medium (8.5 < time < 25.5 min; N = 1152).

To compute average Consistency and Reliability as metrics of signal effectiveness, we adapted MATLAB [23] routines from Bradbury & Vehrencamp [24; Chapter 8 web topics]. In addition, to quantify how effectively begging intensity allows a receiver to infer the underlying deprivation state, we estimated the Bayes decoding accuracy, a standard measure of optimal classification performance [25,26]. Let X denote the deprivation category (the "state") and **S** the observed postural level (the "signal"). From the data, we constructed the empirical joint distribution P(X,S) and its associated posterior probabilities:

$$P(X = x \mid S = s) = P(X = x, S = s) \,/\, P(S = s)$$

A receiver that uses an optimal deterministic decoding rule will, upon observing signal s, choose the state that is most probable given that signal. This is the maximum-posterior (MAP) rule:

$$\hat{X}(s) = \arg\max_x P(X = x \mid S = s).$$

For any given signal s, the probability that this rule yields a correct inference is:

$$P(\text{correct} \mid S = s) = \max_x P(X = x \mid S = s)$$

Bayes decoding accuracy is the overall probability of a correct inference, averaging across all signal levels according to how often each occurs in the data:

$$Acc_{Bayes} = \Sigma_s\, P(S = s) \times \max_x P(X = x \mid S = s)$$

This metric represents the long-run success rate of an ideal receiver using the most informative possible decision rule. High values indicate little overlap between the deprivation distributions associated with different signal levels, whereas low values indicate substantial overlap and therefore reduced discriminability.

Because raw decoding accuracy depends on the marginal distribution of states (e.g., accuracy is inflated if one state is much more common than others), we expressed performance relative to a chance baseline. A naïve receiver that ignores the signal entirely and always chooses the most frequent state would achieve:

$$\text{Acc}_{\text{chance}} = \max_x P(X = x)$$

We then computed the normalised decoding efficiency (NDE) as:

$$\text{NDE} = (\text{Acc}_{\text{Bayes}} - \text{Acc}_{\text{chance}}) \,/\, (1 - \text{Acc}_{\text{chance}})$$

This index measures the proportional improvement that an optimal receiver achieves over the chance strategy. Values of 0 indicate that the signal provides no useful information about deprivation, while values of 1 indicate perfect state discrimination. Intermediate values quantify how much of the maximum possible improvement is realised by the observed coding system.

References

1. Kilner RM. 2000 Multiple nestling begging signals: reed warbler chicks and cuckoo tricks. In: *Animal signals: signalling and signal design in animal communication* (eds Y Espmark, T Amundsen, G Rosenquist), pp. 353–364. Trondheim, Norway: Tapir Academic Press.
2. Krause ET, Honarmand M, Naguib M. 2011 Zebra finch nestlings beg more under better nutritional conditions. *Behaviour* **148**, 1239–1255. (doi:10.1163/000579511X600619)
3. Wright J, Hinde C, Fazey I, Both C. 2002 Begging signals more than just short-term need: Cryptic effects of brood size in the pied flycatcher (*Ficedula hypoleuca*). *Behav. Ecol. Sociobiol.* **52**, 74–83. (doi:10.1007/s00265-002-0478-y)
4. Clark AB. 2002 Appetite and the subjectivity of nestling hunger. In *The evolution of begging: competition, cooperation and communication* (eds J Wright, ML Leonard), pp. 173–198. Dordrecht, Netherlands: Kluwer Academic Publishers. (doi:10.1007/0-306-47660-6_10)
5. Weathers WW. 1996 Energetics of postnatal growth. In *Avian energetics and nutritional ecology* (eds C Carey), pp. 461–496. New York: Chapman and Hall.
6. Moreno-Rueda G, Redondo T. 2012 Benefits of extra begging fail to compensate for immunological costs in southern shrike (*Lanius meridionalis*) nestlings. *PLoS One* **7**, e44647. (doi:10.1371/journal.pone.0044647)
7. Specht R. 2002 *Avisoft-saslab pro: Sound analysis and synthesis laboratory*. Avisoft Bioacoustics, Berlin.
8. Fristrup KM, Watkins WA, Wiebe PH. 1992 *Characterizing acoustic features of marine animal sounds*. Woods Hole Oceanographic Institution.
9. Smith HG, Montgomerie R. 1991 Nestling American robins compete with siblings by begging. *Behav. Ecol. Sociobiol.* **29**, 307–312. (doi:10.1007/BF00163989)
10. Parejo-Pulido D, Pérez-Rodríguez L, Abril-Colón I, Potti J, Redondo T. 2023 Passive and active parental food allocation in a songbird. *Behav. Ecol.* **34**, 729–740. (doi:10.1093/beheco/arad043)
11. Zuur AF, Ieno EN, Walker NJ, Saveliev AA, Smith GM. 2009 *Mixed effects models and extensions in ecology with R*. New York: Springer.

12. Bates D, Mächler M, Bolker B, Walker S. 2015. Fitting Linear Mixed-Effects Models Using lme4. *J. Stat. Softw.* **67**, 1–48. doi:10.18637/jss.v067.i01)
13. Christensen RHB. 2022 ordinal - Regression Models for Ordinal Data. R package version 2022.11-16. https://CRAN.R-project.org/package=ordinal
14. Barr DJ, Levy R, Scheepers C, Tily HJ. 2013 Random effects structure for confirmatory hypothesis testing: Keep it maximal. *J. Mem. Lang.* **68**, 255–278. (doi:10.1016/j.jml.2012.11.001)
15. Bartoń K. 2023 MuMIn: Multi-Model Inference. R package version 1.47.5. (doi:10.32614/CRAN.package.MuMIn)
16. Lüdecke D, Ben-Shachar MS, Patil I, Waggoner P, Makowski D. 2021 performance: An R package for assessment, comparison and testing of statistical models. *J. Open Source Softw.* **6**, 3139. (doi:10.21105/joss.03139)
17. Hartig F. 2022 *DHARMa: Residual Diagnostics for Hierarchical (Multi-Level / Mixed) Regression Models*. R package version 0.4.6. (doi:https://doi.org/10.32614/CRAN.package.DHARMa)
18. Benjamini Y, Hochberg Y. 1995 Controlling the false discovery rate: A practical and powerful approach to multiple testing. *J. R. Stat. Soc. B* **57**, 289–300. (doi:10.1111/j.2517-6161.1995.tb02031.x)
19. Storey JD, Tibshirani R. 2003 Statistical significance for genomewide studies. *Proc. Natl. Acad. Sci. USA* **100**, 9440–9445. (doi:10.1073/pnas.1530509100)
20. Verhoeven KJF, Simonsen KL, McIntyre LM. 2005 Implementing false discovery rate control: increasing your power. *Oikos* **108**, 643–647. (doi:10.1111/j.0030-1299.2005.13727.x)
21. Krzywinski M, Altman N. 2014 Points of significance: Comparing samples-part II. *Nat. Methods* **11**, 355–356. (doi:https://doi.org/10.1038/nmeth.2900)
22. Wickham H, François R, Henry L, Müller K, Vaughan D. 2023 *dplyr: A grammar of data manipulation*. R package version 1.1.4. (doi:10.32614/CRAN.package.dplyr)
23. The MathWorks Inc. 2010 *MATLAB version: 7.11.0 (R2010b), Natick, Massachusetts: The MathWorks Inc.* https://www.mathworks.com
24. Bradbury JW, Vehrencamp SL. 2011 *Principles of animal communication*. Sunderland, MA: Sinauer Associates.
25. Hart PE, Stork DG, Duda RO. 2001. *Pattern Classification*. *2nd ed*. Hoboken: Wiley.
26. Bishop CM, Nasrabadi NM. 2006. *Pattern Recognition and Machine Learning*. New York: Springer.

Supplementary Tables

Table S1. Loadings and variance explained by the first principal component (PC1) for peak frequency, minimum and maximum frequencies, and call entropy.

	Peak	Peak (peakhold mode)	Maximum entire element	Mean entire element	Variance explained (%)
**Peak frequency PC1**	0.55	0.55	0.41	0.49	71.9
**Minimum frequency PC1**	0.50	0.47	0.48	0.55	69.3
**Maximum frequency PC1**	-0.43	-0.52	-0.55	-0.49	64.0
**Call entropy PC1**	0.47	0.51	0.48	0.53	71.9

Table S2. Candidate random structures tested for mixed models ordered by complexity

Random effects structure	Explanation
**Begging in natural broods**	
(1 | Nest) + (1 | Nest:Chick) + (1 | Nest:Sequence)	Nested (Nest/Sequence and Nest/Chick) and crossed (Sequence:Chick) random intercepts only
(1 | Nest) + (0 + deprivation time and/or prey size | Nest:Chick) + (1| Nest:Sequence)	Random slopes only
(0 + deprivation time and/or prey size | Nest:Chick) + (1 | Nest/Chick) + (1 | Nest:Sequence)	Uncorrelated random intercepts and slopes
(1 | Nest) + (deprivation time and/or prey size | Nest:Chick) + (1 | Nest:Sequence)	Correlated random intercepts and slopes
**Probability of feeding in natural broods**	
(1 | Nest) + (1 | Nest:Parent)	Nested (Nest/Sequence and Nest/Parent) and crossed (Sequence:Parent) random intercepts only
(1 | Nest) + (0 + begging behavior | Nest:Parent)	Random slopes only
(1 | Nest) + (0 + begging behavior | Nest:Parent) + (1 | Nest:Parent)	Uncorrelated random intercepts and slopes
(1 | Nest) + (begging behavior | Nest:Parent)	Correlated random intercepts and slopes
**Begging variables in the laboratory experiment**	
(1 | Nest/Chick)	Nested random intercepts only[1]
(0 + deprivation time | Nest/Chick)	Random slopes only[2]
(0 + deprivation time | Nest/Chick) + (1 | Nest/Chick)	Uncorrelated random intercepts and slopes[3]
(deprivation time | Nest/Chick)	Correlated random intercepts and slopes[4]

[1] Chicks and broods may differ in their absolute mean begging values, but they all co-vary similarly with deprivation time

[2] Chicks and broods have a similar mean begging value. Chicks may differ in how a begging variable covaries with deprivation time

[3] Chicks and broods may differ in absolute mean begging values. Chicks may differ in how begging covaries with deprivation time but the slope of this relationship is independent from absolute mean values

[4] Chicks and broods may differ in absolute mean begging values. Chicks may differ in how begging covaries with deprivation time and the slope of this relationship covaries with absolute mean values

Table S3. Optimal random-effects structures for models of natural brood observations, by response variable, and variance explained by each random effect.

	Variance explained				
	Chick		Nest	Visit	
**Optimal structure**	**Intercept**	**Slope**	**Intercept**	**Intercept**	**Residual**
**Maximum postural intensity**<br>(1 | Nest) + (prey size + deprivation time | Nest:Chick) + (1 | Nest:Sequence)	0.23	0.22<br>0.17	0.59	1.82	*
**Begging order**<br>(0+prey size|Nest:Chick) + (0+ deprivation time |Nest:Chick) + (1|Nest:Chick)	0.37	0.10<br>0.04			*
**Probability of feeding**<br>(1 | Nest) + (0 + postural intensity + begging order + spatial profitability | Nest:Parent)		<0.01<br>0.03<br>0.12	0.02	-	*

* Obtaining an appropriate estimate for residual variance is not straightforward for generalized mixed linear models (e.g. binomial, as in this case) and ordinal regression models (Nakagawa & Schielzeth, 2013).

Table S4. Optimal random-effects structures for laboratory models, by response variable, and variance explained by each random effect.

	Variance explained				
	Chick		Nest		
**Optimal structure**	**Intercept**	**Slope**	**Intercept**	**Slope**	**Residual**
**Mean postural intensity**<br>(0 + deprivation time |Nest/Chick) + (1 | Nest/Chick)	0.06	0.01	0.07	0.01	0.12
**Maximum postural intensity**<br>(0 + deprivation time |Nest/Chick) + (1 | Nest/Chick)	2.89	0.60	1.59	0.19	*
**Duration of postural display**<br>(1 | Nest/Chick)	2.76		3.65		12.0
**Latency to gape**<br>(0 + deprivation time | Nest/Chick)		<0.01		<0.01	<0.01
**Time displaying at maximum**<br>(1 | Nest/Chick)	<0.01		1.97		12.5
**Probability of reaching level 4**<br>(deprivation time | Nest/Chick)	2.66	0.38	0.75	0.89	*
**Probability of reaching level 3**<br>(1 | Nest/Chick)	0.04		1.34		*
**Probability of reaching level 2**<br>(0 + deprivation time | Nest/Chick) + (1 | Nest/Chick)	2.97	1.89	1.05	0.08	*
**Probability of reaching level 1**<br>(0 + deprivation time | Nest/Chick) + (1 | Nest/Chick)	33.1	24.0	<0.01	0.14	*

<table>
<tr><td></td><td></td><td></td><td></td><td></td><td></td></tr>
<tr><td>Time displaying at level 4 (all displays)
(deprivation time | Nest/Chick)</td><td>0.10</td><td>0.03</td><td>0.03</td><td>0.02</td><td>0.16</td></tr>
<tr><td>Time displaying at level 4 as maximum
(1 | Nest/Chick)</td><td>0.61</td><td></td><td>0.05</td><td></td><td>2.15</td></tr>
<tr><td>Time displaying at level 3 (all displays)
(0 + deprivation time |Nest/Chick) + (1 | Nest/ Chick)</td><td>0.18</td><td>0.08</td><td>0.20</td><td><0.01</td><td>0.44</td></tr>
<tr><td>Time displaying at level 3 as maximum
(1 | Nest/ Chick)</td><td><0.001</td><td></td><td>1.10</td><td></td><td>7.01</td></tr>
<tr><td>Time displaying at level 2 (all displays)
(0 + deprivation time |Nest/Chick) + (1 | Nest/ Chick)</td><td>1.64</td><td>1.36</td><td>1.30</td><td>0.05</td><td>12.0</td></tr>
<tr><td>Time displaying at level 2 as maximum
(1 | Nest/Chick)</td><td>4.46</td><td></td><td>0.34</td><td></td><td>5.65</td></tr>
<tr><td>Time displaying at level 1 (all displays)
(0 + deprivation time |Nest/Chick) + (1 | Nest/ Chick)</td><td>0.08</td><td>0.02</td><td>0.09</td><td>0.02</td><td>0.29</td></tr>
<tr><td>Calling rate
(1 | Nest/Chick)</td><td>0.06</td><td></td><td>0.01</td><td></td><td>0.02</td></tr>
<tr><td>Duration of vocal bout
(1 | Nest/Chick)</td><td>2.86</td><td></td><td>2.90</td><td></td><td>5.61</td></tr>
<tr><td>Latency to call
(0 + deprivation time | Nest/Chick)</td><td></td><td><0.01</td><td></td><td><0.01</td><td><0.01</td></tr>
<tr><td>Call duration
(0 + deprivation time | Nest/Chick) + (1 | Nest/Chick)</td><td><0.01</td><td><0.01</td><td><0.01</td><td><0.01</td><td><0.01</td></tr>
<tr><td>Distance from start to peak
(0 + deprivation time | Nest/Chick) + (1 | Nest/Chick)</td><td><0.01</td><td><0.01</td><td><0.01</td><td><0.01</td><td><0.01</td></tr>
<tr><td>Peak frequency PC1
(0 + deprivation time | Nest/Chick) + (1 | Nest/Chick)</td><td>1.34</td><td>0.09</td><td>0.57</td><td>0.02</td><td>0.90</td></tr>
<tr><td>Minimum frequency PC1
(0 + deprivation time | Nest/Chick) + (1 | Nest/Chick)</td><td>1.59</td><td>0.06</td><td>0.49</td><td>0.03</td><td>0.59</td></tr>
<tr><td>Maximum frequency PC1
(0 + deprivation time | Nest/Chick) + (1 | Nest/Chick)</td><td>1.46</td><td>0.11</td><td>0.24</td><td>0.01</td><td>0.74</td></tr>
<tr><td>Call entropy PC1
(0 + deprivation time | Nest/Chick) + (1 | Nest/Chick)</td><td>1.00</td><td>0.09</td><td>1.01</td><td><0.01</td><td>0.68</td></tr>
<tr><td>SD of the frequency P25
(0 + deprivation time | Nest/Chick) + (1 | Nest/Chick)</td><td><0.01</td><td><0.01</td><td><0.01</td><td><0.01</td><td><0.01</td></tr>
<tr><td>SD of the frequency P75
(deprivation time | Nest/Chick)</td><td><0.01</td><td><0.01</td><td><0.01</td><td><0.01</td><td><0.01</td></tr>
</table>

* Obtaining an appropriate estimate for residual variance is not straightforward for generalized mixed linear models (e.g. binomial, as in this case) and ordinal regression models (Nakagawa & Schielzeth, 2013).

Table S5. Parameter estimates (± s.e.), F-values, degrees of freedom, t-values and *P*-values for models testing effects of categorical deprivation time on the duration of decomposed postural intensity levels in the laboratory. Durations are analysed across all displays and when a given level was reached as the maximum. Reference categories are 30 minutes (deprivation time) and male (sex).

	**Estimate**	***SE***	***F***	**df**	***t***	***P***
**Time at level 3 (all displays)**						
Intercept	0.61	0.14			4.19	<0.001
Deprivation time			4.18	2,23		0.028
60 min	0.26	0.14			1.88	0.073
90 min	0.44	0.15			2.95	0.007
**Time at level 3 at maximum**						
Intercept	6.21	0.76			8.21	<0.001
Deprivation time			0.26	2,45		0.771
60 min	0.73	1.03			0.72	0.478
90 min	0.41	1.01			0.41	0.685
**Time at level 2 (all displays)**						
Intercept	6.00	0.47			12.9	<0.001
Body mass	0.72	0.34	4.50	1,43	2.12	0.040
Deprivation time			0.56	2,248		0.573
60 min	-0.53	0.52			-1.02	0.309
90 min	-0.39	0.52			-0.75	0.454
**Time at level 2 at maximum**						
Intercept	8.23	0.49			16.7	<0.001
Deprivation time			2.39	2,112		0.096
60 min	0.44	0.52			0.85	0.398
90 min	1.27	0.58			2.18	0.031

[4] Predictors other than Deprivation time (Sex, body mass, brood size, date and stimulus) and their interactions that failed to improve model fit are not depicted.

Table S6. Parameter estimates (± s.e.) for the effect of deprivation time on begging variables from models refitted without random slopes. Uncorrected and Benjamini–Hochberg-corrected P-values are reported for the effect of time since the last feeding.

Variable	Interaction	Estimate	SE	*P*	Corrected *P*
**VISUAL COMPONENTS**					
Mean postural intensity		0.15	0.02	<0.001	<0.001
Maximum postural intensity		0.88	0.14	0.004	0.011
Latency to gape		-0.01	<0.01	0.028	0.035
Probability of reaching level 4	Date	0.09	0.03	0.001	0.003
	Brood size	-0.07	0.03	0.008	0.022
Probability of reaching level 2	Body mass	-0.37	0.15	0.014	0.028
Probability of reaching level 1		-1.52	0.66	0.022	0.035
Time displaying at level 4 (all displays)	Sex	-0.17	0.05	0.001	0.003
Time displaying at level 3 (all displays)		0.18	0.04	<0.001	<0.001
Time displaying at level 2 (all displays)		-0.16	0.21	0.453	0.518
Time displaying at level 1 (all displays)		-0.06	0.03	0.090	0.119
**ACOUSTIC COMPONENTS**					
Latency to call		<-0.01	<0.01	0.372	0.409
Call duration		0.01	<0.01	<0.001	<0.001
Distance to peak		<0.01	<0.01	0.042	0.058
Peak frequency PC1	Brood size	-0.11	0.02	<0.001	<0.001
Minimum frequency PC1		0.16	0.02	<0.001	<0.001
Maximum frequency PC1		-0.07	0.02	0.001	0.001
Call entropy PC1		0.01	0.02	0.668	0.668
SD of the frequency P25		<-0.01	<0.01	<0.001	0.001
SD of the frequency P75		<-0.01	<0.01	<0.001	<0.001

Supplementary Figures

Figure S1. Oscillogram (top), spectrogram in false colour (centre) and average amplitude spectrum (left) of a laboratory begging bout. The playback stimulus ends at approximately 0.5 s.

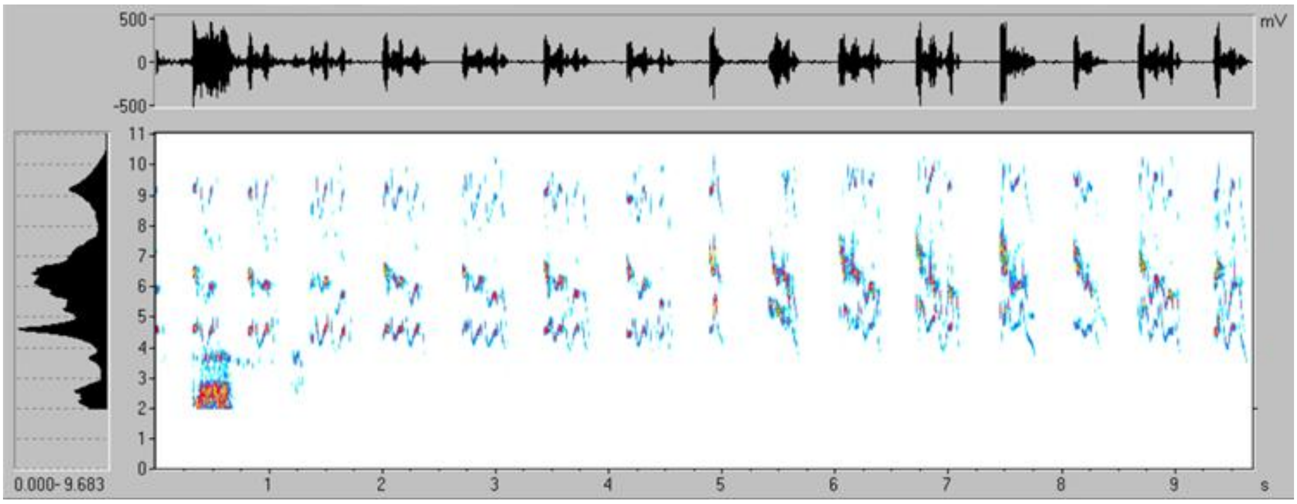

Figure S2. Kernel density distributions of deprivation time by maximum postural intensity in natural broods

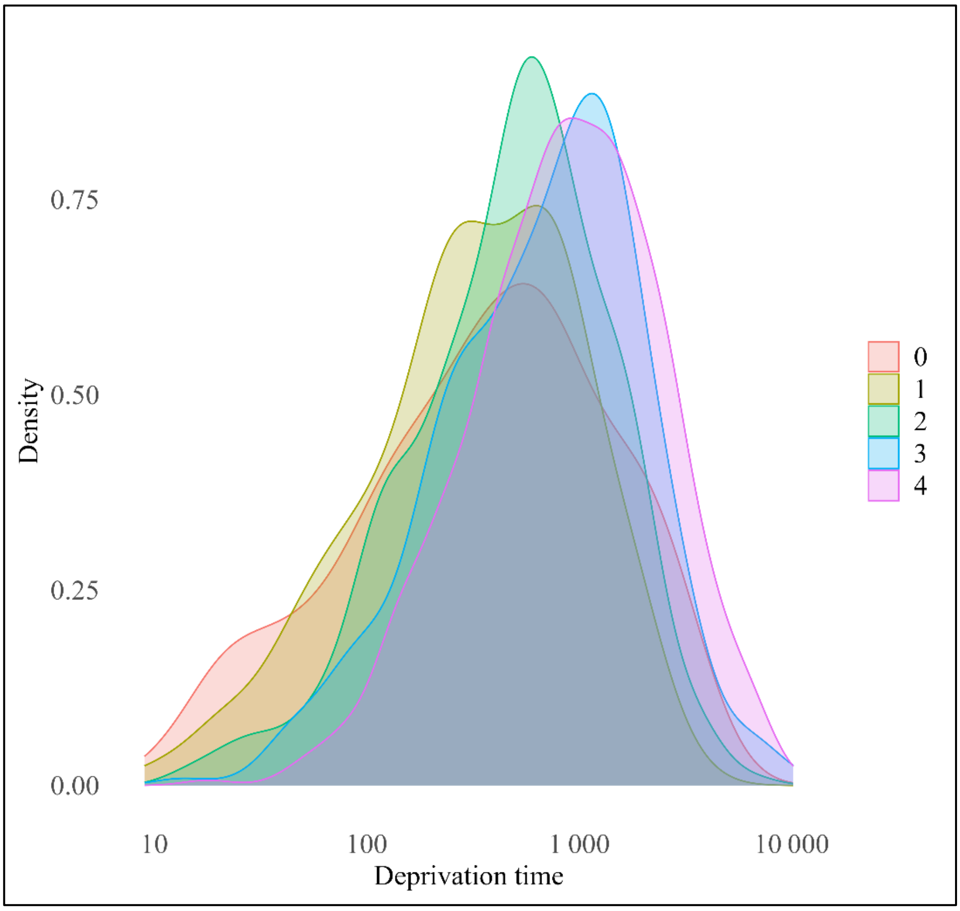

Figure S3. Combined effects of prey size at the previous visit and time since the last feeding on standardised begging order in natural broods (1 = first to beg). Prey size is shown in quartiles for visualisation. Lines represent fitted values with 95% confidence intervals (grey bands).

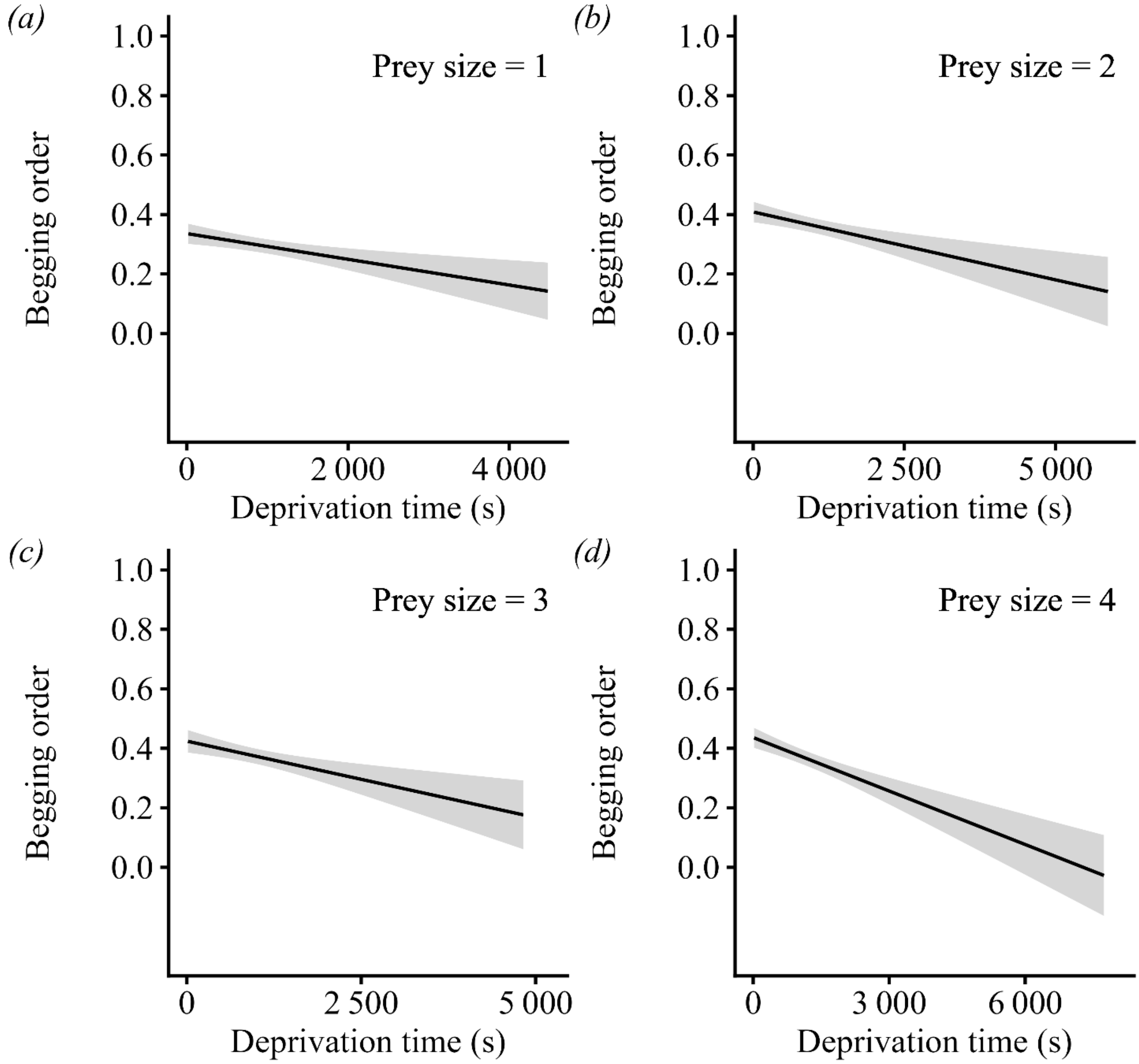

Figure S4. Combined effects of standardised begging order (1 = first to beg) and postural intensity on the probability of being fed. Begging order is shown in quartiles for visualisation. Lines represent fitted values with 95% confidence intervals (grey bands).

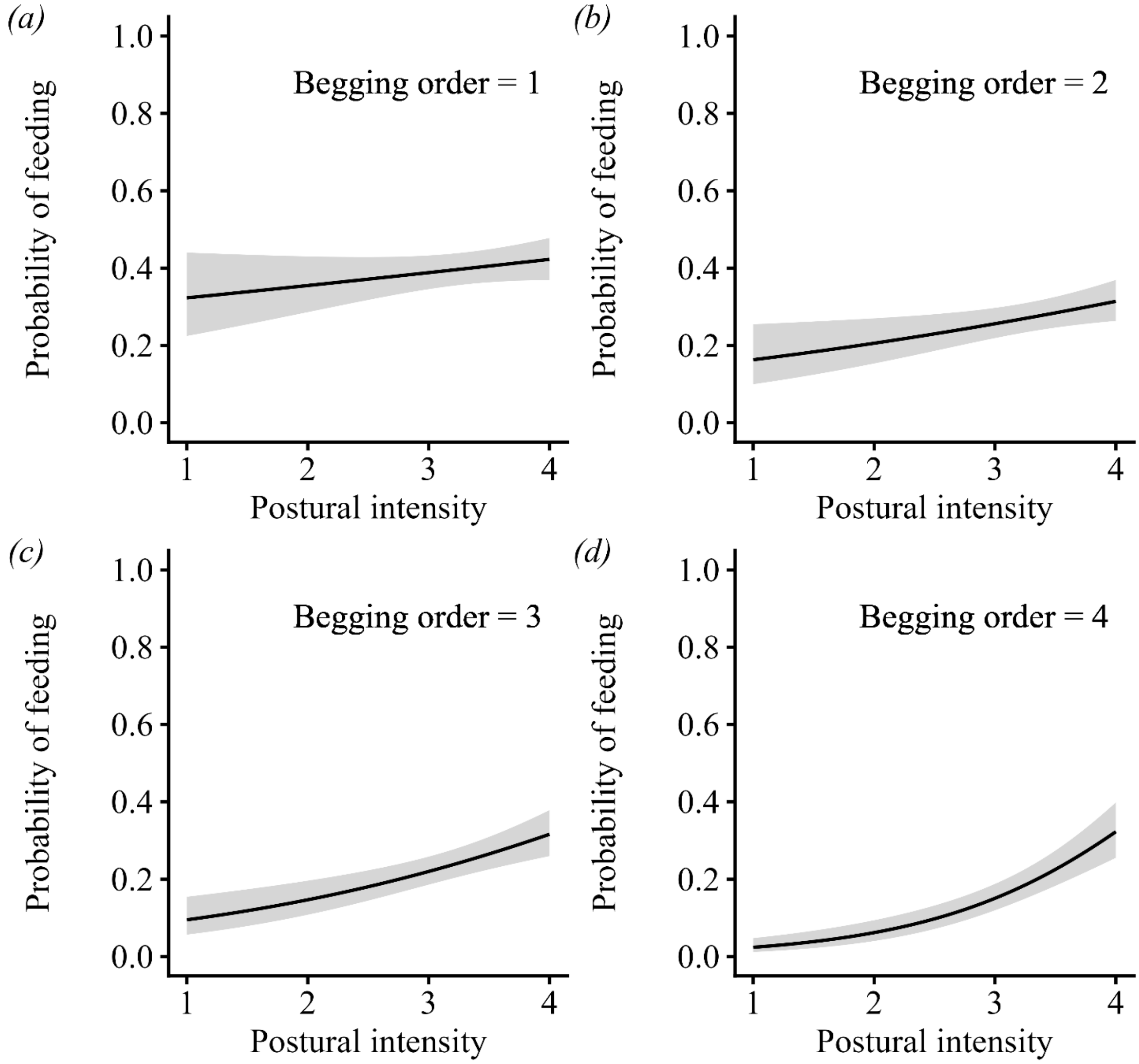

Figure S5. Effects of deprivation time and brood size on total postural display duration. Points show raw values; lines represent fitted regressions with 95% confidence intervals (grey bands).

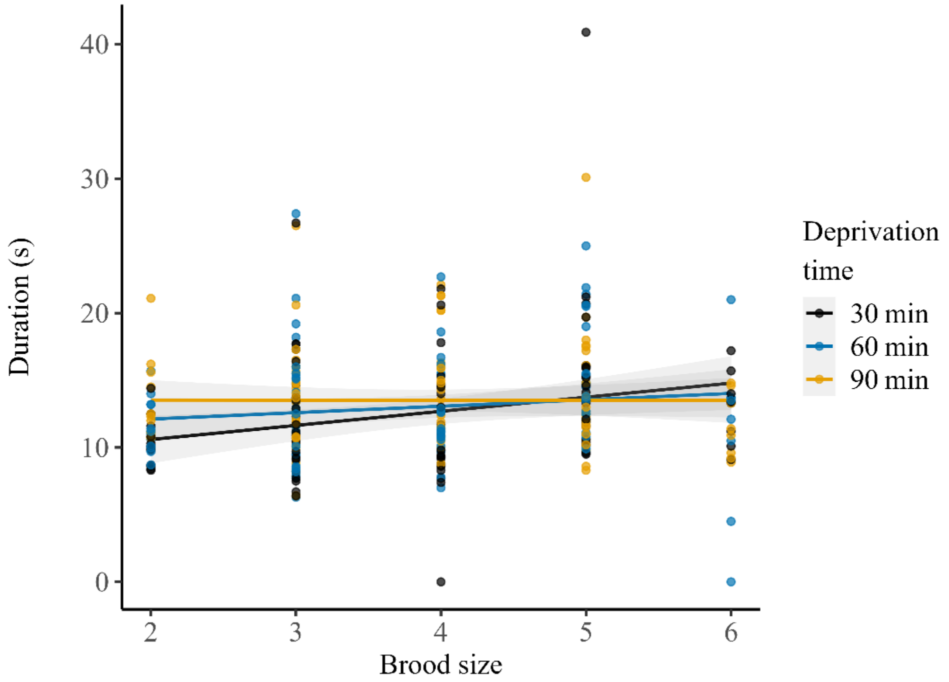

Figure S6. Receiver-based decoding of deprivation state from laboratory begging bouts using postural measures of latency and display duration (instead of acoustic measures, as in Figure 5). Lines show mean (± s.d.) grouped K-fold cross-validated random-forest accuracy as components (1–14) are added sequentially during a bout. Components are ordered by decreasing semi-partial R^2 (a), rank-based uncertainty reduction (b) and absolute-scale uncertainty reduction (c). Postural latency enters first, maximum postural intensity second, and total postural display duration last.

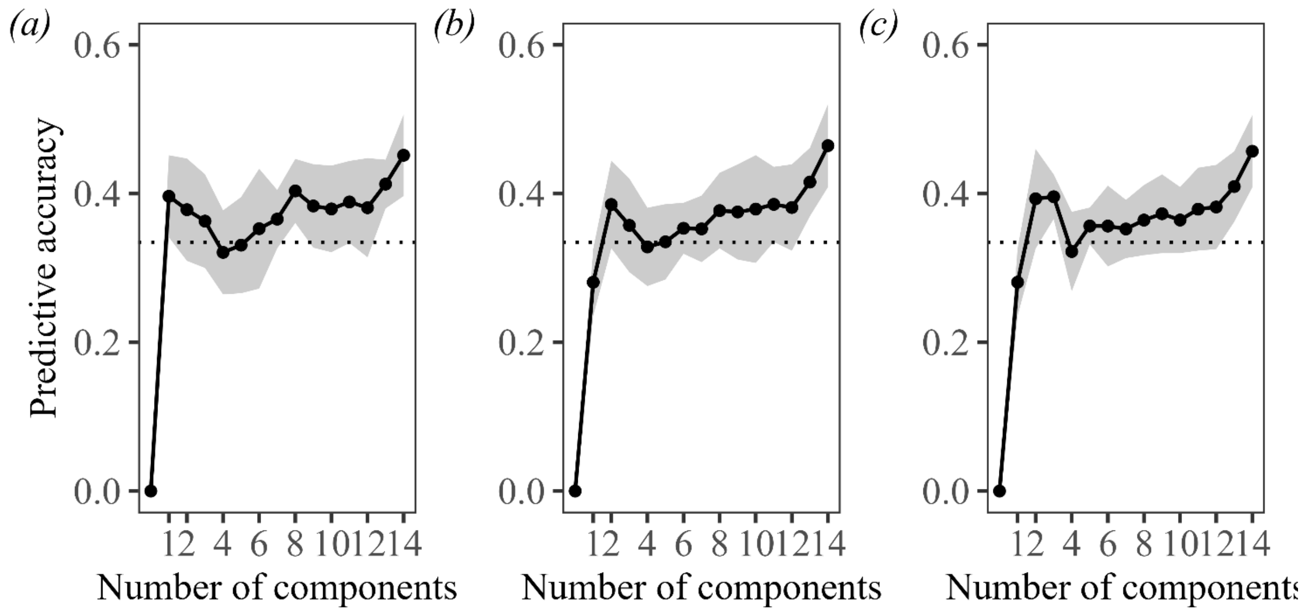

Supplement: Supplementary file 1 [file rsos.260413.zip › rsos.260413/rsos260413_si_001.pdf]
